# Circular RNA APP contributes to Alzheimer’s disease pathogenesis by modulating microglial polarization via miR-1906/CLIC1 axis

**DOI:** 10.1186/s13195-025-01698-7

**Published:** 2025-02-14

**Authors:** Deng-Pan Wu, Yan-Su Wei, Li-Xiang Hou, Yu-Xuan Du, Qiu-Qing Yan, Ling-Ling Liu, Yuan-Dan Zhao, Ru-Yu Yan, Chao Yu, Zhen-Guo Zhong, Jin-Lan Huang

**Affiliations:** 1 Jiangsu Key Laboratory of New Drug Research and Clinical Pharmacy, Pharmacy School, Xuzhou Medical University, Xuzhou, 221004 Jiangsu China; 2 Xuzhou Ruihu Health Management Consulting Co., Ltd, Xuzhou, 221002 Jiangsu China; 3 Department of Psychiatry, Chongqing Medical University, Chongqing, 400016 China; 4 School of Basic Medicine, Xuzhou Medical University, Xuzhou, 221004 Jiangsu China; 5 Scientific Research Center of Traditional Chinese Medicine, Guangxi University of Chinese Medicine, Nanning, 530200 Guangxi China

**Keywords:** circRNA, Alzheimer’s disease, Microglial polarization, miR-1906, CLIC1

## Abstract

**Background:**

Abnormal microglial polarization phenotypes contribute to the pathogenesis of Alzheimer’s disease (AD). Circular RNAs (circRNAs) have garnered increasing attention due to their significant roles in human diseases. Although research has demonstrated differential expression of circRNAs in AD, their specific functions in AD pathogenesis remain largely unexplored.

**Methods:**

CircRNA microarray was performed to identify differentially expressed circRNAs in the hippocampus of APP/PS1 and WT mice. The stability of circAPP was assessed via RNase R treatment assay. CircAPP downstream targets miR-1906 and chloride intracellular channel 1 (CLIC1) were identified using bioinformatics and proteomics, respectively. RT-PCR assay was conducted to detect the expression of circAPP, miR-1906 and CLIC1. Morris water maze (MWM) test, passive avoidance test and novel object recognition task were used to detect cognitive function of APP/PS1 mice. Microglial M1/M2 polarization and AD pathology were assessed using Western blot, flow cytometry and Golgi staining assays. CLIC1 expression and channel activity were evaluated using Western blot and functional chloride channel assays, respectively. The subcellular location of circAPP was assessed via FISH and RT-PCR assays. RNA pull-down assay was performed to detect the interaction of miR-1906 with circAPP and 3’ untranslated region (3’UTR) of CLIC1 mRNA.

**Results:**

In this study, we identified a novel circRNA, named circAPP, that is encoded by amyloid precursor protein (APP) and is implicated in AD. CircAPP is a stable circRNA that was upregulated in Aβ-treated microglial cells and the hippocampus of APP/PS1 mice. Downregulation of circAPP or CLIC1, or overexpression of miR-1906 in microglia modulated microglial M1/M2 polarization in Aβ-treated microglial cells and the hippocampus of APP/PS1 mice, and improved AD pathology and the cognitive function of APP/PS1 mice. Further results revealed that circAPP was mainly distributed in the cytoplasm, and circAPP could regulate CLIC1 expression and channel activity by interacting with miR-1906 and affecting miR-1906 expression, thereby regulating microglial polarization in AD.

**Conclusions:**

Taken together, our study elucidates the regulatory role of circAPP in AD microglial polarization via miR-1906/CLIC1 axis, and suggests that circAPP may act as a critical player in AD pathogenesis and represent a promising therapeutic target for AD.

**Supplementary Information:**

The online version contains supplementary material available at 10.1186/s13195-025-01698-7.

## Background

Alzheimer’s disease (AD), the primary cause of dementia, is pathologically characterized by the presence of senile plaques due to excessive amyloid β (Aβ) aggregation, neurofibrillary tangles composed of hyperphosphorylated tau, and synaptic dysfunction in the hippocampus and cortex of patients [1]. Microglia, the resident immune effector cells in the brain, have been proposed as critical participants in AD [2]. Microglia can dynamically polarize into M1 (proinflammatory) and M2 (anti-inflammatory) phenotypes [3]. During the M1 phenotype, microglia appear to express phenotypic markers such as cluster of differentiation (CD)16 and produce proinflammatory cytokines such as tumor necrosis factor-α (TNF-α) and interleukin-1β (IL-1β), whereas microglia in the M2 phenotype express phenotypic markers such as arginase (ARG)-1, enable phagocytosis and secrete anti-inflammatory cytokines such as IL-10 [3, 4]. Aberrant stimuli such as Aβ aggregates activate the M1 phenotype, leading to the sustained release of proinflammatory cytokines, whereas the M2 phenotype is strongly inhibited during AD progression, thereby weakening its effects on Aβ phagocytosis and immunosuppression [5–8]. The imbalance of microglial polarization contributes to a vicious cycle between chronic neuroinflammation and AD pathology, which ultimately causes synaptic injury and cognitive decline [9, 10]. Thus, improving aberrant microglial polarization may represent a promising therapeutic strategy for dampening AD progression [2, 3].

CircRNAs, a class of novel RNAs, lack a 5’ cap and a 3’ polyadenylated tail [11]. Unlike linear RNAs, circRNAs are more stable because of their covalently closed loop [11]. CircRNAs were considered byproducts of splice errors until their function as microRNA (miRNA) sponges was revealed [12]. Recently, circRNAs have attracted increasing attention owing to their crucial roles in human diseases, including cancer, cardiovascular diseases, diabetes mellitus, and neurological diseases [13–16]. Studies have shown that circRNAs are highly abundant in mammalian brains and tend to accumulate in the brain during aging [17–19], indicating that they may be involved in age-related neurodegenerative diseases, including AD [20]. Indeed, several lines of evidence have shown that circRNAs are differentially expressed in AD animal models and patients compared with healthy controls [21, 22]. However, the precise roles of most circRNAs in the progression of AD remain unclear. Therefore, validating the critical functions and regulatory mechanisms of key circRNAs is important for developing novel therapeutic strategies against AD.

In this study, we identified a circRNA derived from amyloid precursor protein (APP) (circAPP) that was upregulated in the hippocampus of APP/PS1 mice compared with healthy controls. Additionally, circAPP was upregulated in Aβ-treated microglia. CircAPP knockdown in microglia modulated microglial M1/M2 polarization in vitro and in vivo, and improved AD pathology and cognitive function in vivo. Further results revealed that circAPP could regulate the expression and channel activity of chloride intracellular channel 1 (CLIC1) by interacting with miR-1906 and affecting miR-1906 expression, leading to abnormal microglial polarization in AD. Taken together, these results reveal a novel regulatory role of circAPP in modulating AD microglial polarization via miR-1906/CLIC1 axis, indicating that circAPP may act as a crucial player in AD pathogenesis and represent a promising target for AD treatment.

## Methods

### Mice and ethics statement

Male APP/PS1 transgenic mice expressing mouse/human APP695 Swedish mutations and human PS1 mutations and male wild-type (WT) C57BL/6J mice were purchased from Changzhou Cavens Model Animal Co., Ltd. (Changzhou, China). The mice were housed under constant humidity and temperature with a 12:12 h light-dark cycle and allowed to tap food and water freely. Animal care and experimental procedures were approved by the animal welfare committee of Xuzhou Medical University.

### Cell culture, transfection and Aβ treatment

Primary neuron and glial cultures were prepared from the hippocampus of 1-day-old C57BL/6J mice as previously described with modifications [23]. Briefly, the hippocampus was dissected and incubated in trypsin. Neurons were dissociated and plated on plates precoated with poly-L-lysine containing neurobasal medium (Thermo Fisher) and 2% B27 supplement (Thermo Fisher) in humidified air (37 °C, 5% CO_2_). For glial cell culture, after the dissected hippocampus was digested with trypsin, the cells were placed on poly-L-lysine-precoated plates containing Dulbecco’s modified Eagle’s medium (DMEM) (Dalian Meilun Biotechnology Co., Ltd., Dalian, China) supplemented with 10% (v/v) FBS (ExCell Bio, Suzhou, China) and 1% (v/v) penicillin/streptomycin in humidified air containing 5% CO_2_ at 37 °C for 7 days. The glial cells were harvested via trypsinization.

BV-2 mouse microglia cells, C8-D1A mouse astrocytic cells, and Neuro-2a (N2a) mouse neuronal cells (ATCC) were cultured in DMEM (Sangon Biotech, Shanghai, China) supplemented with 10% (v/v) FBS (ExCell Bio, Suzhou, China) and 1% (v/v) penicillin/streptomycin in humidified air containing 5% CO_2_ at 37 °C. BV-2 cells were transfected with either circControl shRNA and circAPP shRNA lentiviruses (LVs) (GenePharma, Suzhou, China), miR-control, miR-1906 (General Biol, Chuzhou, China) and CLIC1 (GenePharma, Suzhou, China) plasmids, or control shRNA, miR-1906 shRNA and CLIC1 shRNA plasmids (GenePharma, Suzhou, China). The sequences of the shRNAs targeting circAPP and CLIC1 are presented in Supplementary Table S1. Transfections were conducted by adding lentiviruses or plasmids to the growth medium according to the manufacturer’s instructions. Stable cell lines were screened with hygromycin, blasticidin, G418, or puromycin. Primary neurons and glial cells, as well as BV-2, C8-D1A, N2a cells and stable cell lines, were treated with 40 µM Aβ_25−35_ (GenicBio, Shanghai, China) for 24 h, after which the cells were subsequently collected for RT-PCR, Western blot and functional chloride channel assays.

### CircRNA microarray

Three hippocampal samples from APP/PS1 mice and three other hippocampal samples from WT mice were screened via Mouse circRNA Microarray (8 × 15 K, Arraystar, Rockville, MD, USA). In brief, linear RNAs were removed with Rnase R to enrich circRNAs. Then, the enriched circRNAs were amplified and transcribed into fluorescent cRNA using a random priming method (Arraystar Super RNA Labeling Kit; Arraystar). The labeled cRNAs were hybridized onto the Arraystar Mouse circRNA Microarray V2. After the slides were washed, the arrays were scanned by the Agilent Scanner G2505C. Finally, Agilent Feature Extraction software (version 11.0.1.1) was utilized to analyze acquired array images. Quantile normalization and subsequent data processing were performed using R software.

### Real-time PCR and RNase R treatment assays

Total RNA was extracted using TRIzol reagent (Mei5 Biotech., Beijing, China). After reverse transcription, samples were mixed with SYBR Green Master Mix (Servicebio Technology, Wuhan, China) and primers via a Roche 480 LightCycler detection system. The primer sequences are listed in Supplementary Table S1. The relative expression of genes was calculated via the 2^−△△CT^ method. The expression of ACTB and small nuclear U6 was used to normalize the relative cytoplasmic and nuclear expression of genes, respectively. For detecting the stability of circAPP, total RNA was treated with RNase R (Guangzhou Geneseed Biotech. Co., Ltd., Guangzhou, China), then RT-PCR assay was performed to analyze the expression of circAPP and ACTB mRNA.

### Hippocampal microinjection of adeno-associated viruses

Hippocampal microinjection of adeno-associated viruses (AAVs) was performed according to a previous study with some modifications [24]. In brief, APP/PS1 mice were fixed after anesthesia. Control and circAPP AAVs as well as microglia-specific control shRNA AAVs (AAV-CX3CR1-shRNA-Con), circAPP shRNA AAVs (AAV-CX3CR1-shRNA-circAPP), CLIC1 shRNA AAVs (AAV-CX3CR1-shRNA-CLIC1), or microglia-specific control AAVs (AAV-CX3CR1-Con) and miR-1906 AAVs (AAV-CX3CR1-Pri-miR-1906) (Obio, Shanghai, China) were injected into the bilateral hippocampus at the following coordinates: 1.5 mm lateral to the midline, 1.94 mm posterior to the bregma, and 1.75 mm below the dura at a rate of 0.1 µL/min. After injection, the needle was kept in place for 10 min to allow diffusion. The sequences of circAPP and CLIC1 shRNA are presented in Supplementary Table S1. After 6 weeks of AAV infection, 3 ~ 4 mice were randomly selected from each group for measuring the efficiency of AAV infection in the hippocampus of mice using RT-PCR assay.

### Morris water maze (MWM) test

The MWM test was used to assess the learning and memory abilities of APP/PS1 mice according to a previous study [24]. Briefly, in the navigation test, the mice were subjected to training trials to locate the hidden platform for 4 days. In the spatial probe test, the platform was removed, and the spatial memory ability of the mice was measured. Since the time spent in the target quadrant (quadrant time) has been reported as a common parameter to evaluate the spatial memory ability of mice in the spatial probe test [25–27], quadrant time was recorded in the spatial probe test.

### Passive avoidance test

The passive avoidance test was performed as previously described [28]. Briefly, on the training day, the mice were allowed to enter the dark compartment, where they were subjected to foot shock (0.2 mA). After 24 h, the memory errors of each mouse were recorded.

### Novel object recognition task

The novel object recognition task was conducted according to a previous study [29]. In brief, the mice were placed in a room where two identical objects were placed. A novel object was placed to replace a familiar object after 24 h. The head entries exploring the novel object (N) and the familiar object (F) were recorded separately, and the percentage of entries on novel objects was calculated according to the following equation: N%=N/N + F.

### Label-free quantitative proteomic analysis

Label-free quantitative proteomic analysis was performed by Aksomics Bioscience Institute (Shanghai, China) to identify differentially expressed proteins in the hippocampus of WT mice overexpressing circAPP. In brief, the samples were lysed in RIPA buffer supplemented with a protease inhibitor cocktail. Proteins were then precipitated using acetone, followed by dissolution, reduction, alkylation, trypsinization, and desalination. The digested peptides were subsequently detected via mass spectrometry (Thermo Fisher Scientific, MA, USA), followed by identification and quantitation of the peptides and proteins.

### Western blot assay

After the protein samples were separated and transferred, primary antibodies against Aβ_42_ (Biolegend, San Diego, USA, dilution: 1:1000), p-tau S396 (ABclone, Wuhan, China, dilution: 1:1000), pro-and cleaved IL-1β (Affinity Biosciences, dilution: 1:1000), TNF-α (Proteintech, Wuhan, China, dilution: 1:1000), IL-10 (Proteintech, Wuhan, China, dilution: 1:800), CD16 (Proteintech, Wuhan, China, dilution: 1:1000), Arg1 (Proteintech, Wuhan, China, dilution: 1:1000), CLIC1 (Proteintech, Wuhan, China, dilution: 1:1000), Iba-1 (Proteintech, Wuhan, China, dilution: 1:1000), Gapdh (Proteintech, Wuhan, China, dilution: 1:3000) and β-actin (ABclonal, Wuhan, China, dilution: 1:8000) were incubated at 4 °C overnight. Then, the sections were incubated with IRDye-purified secondary antibody (LI-COR Biosciences, dilution: 1:10000) and immunopositive bands were visualized at Ex/Em = 778 nm/795 nm.

### RNA pull-down assay

The RNA pull-down assay was performed according to previous studies with some modifications [30, 31]. Briefly, biotinylated probes for circAPP and miR-1906 (General Biology Co., Ltd., Chuzhou, China) were designed to bind to the circAPP junctional site and miR-1906, respectively. The probe sequences are listed in Supplementary Table S1. To detect the interaction between circAPP and miR-1906, BV-2 cells overexpressing circAPP were harvested and lysed. The circAPP probe was incubated with streptavidin magnetic beads (BEAVER Biomedical, Suzhou, China) at 4°C for 2 h. The cell lysates were incubated with beads precoated with a circAPP probe at 4°C overnight. The beads were then washed, and miRNAs interacting with circAPP in the pull-down materials were extracted. Subsequently, circAPP and miR-1906 expression was detected via RT-PCR assay. To measure the interaction between miR-1906 and 3’ untranslated region (3’UTR) of CLIC1 mRNA, lysates from BV-2 cells overexpressing miR-1906 were collected and incubated with beads precoated with the miR-1906 probe at 4 °C overnight. Then, an RT-PCR assay was used to detect the expression of miR-1906 and 3’UTR of CLIC1 mRNA after the beads were washed, and RNAs interacting with miR-1906 were extracted.

### Flow cytometry assay

The ability of BV-2 cells to phagocytose Aβ was detected via flow cytometry. Briefly, BV-2 cells were treated with FITC-labeled Aβ_42_ (GenicBio, Shanghai, China) for 24 h. The cells were collected and resuspended in PBS. Then, the FITC-positive cells were detected via a flow cytometer (Agilent Technologies, Inc., USA) according to the manufacturer’s instructions.

### RNA-fluorescence in situ hybridization (FISH) assay

FAM-labeled probes were specific to circAPP (Servicebio Technology, Wuhan, China), and Cy3-labeled probes were specific to miR-1906 (General Biology Co., Ltd., Chuzhou, China). The signals of the probes were measured via a Fluorescent In Situ Hybridization Kit (Guangzhou RiboBio Co., Ltd., Guangzhou, China) according to the manufacturer’s instructions. Images were captured with a confocal laser scanning microscope (CLSM, Leica STELLARIS 5, Germany).

### Golgi staining assay

The Golgi staining assay was performed via an FD Rapid GolgiStain™ Kit (FD NeuroTechnologies, Inc., Columbia, USA) according to the manufacturer’s instructions. In brief, after the brains were washed with cold PBS, they were immersed in solutions A and B for 14 days and in solution C for 3 days. The brains were subsequently sliced and stained with a staining solution for 10 min at room temperature. The hippocampal synaptic morphology of the mice was photographed via a microscope (Olympus Corporation, Tokyo, Japan). The spine density (number of dendritic spines per µm) of the hippocampal neurons were analyzed using ImageJ software.

### Functional chloride channel assay

CLIC1 channel activity was measured via a functional chloride channel kit (AAT Bioquest, California, USA) according to the manufacturer’s instructions [32]. In brief, after the BV-2 cells were plated, 100 µL of iodine-loading buffer was added, and the mixture was incubated at 37 °C for 5 min. The cells were washed three times with PBS and lysed with lysis buffer. An iodide assay was subsequently performed by adding the Iodide Blue™ sensor and iodide sensor enhance solution to the wells, after which the absorbance was detected at 630 nm. Chloride channel activity was expressed as iodide uptake, which was calculated using the absorbances of different concentrations of iodide solution via nonlinear regression analysis.

### Statistical analysis

The data from each group are presented as means ± SEMs. The data were statistically analyzed via Student’s *t* test and one-way analysis of variance (ANOVA) to compare the differences between two and more than three groups, respectively, via SPSS software for Windows 20.0. *P* < 0.05 was considered to indicate statistical significance.

## Results

### CircAPP is upregulated in the hippocampus of APP/PS1 mice

**Fig. 1:**
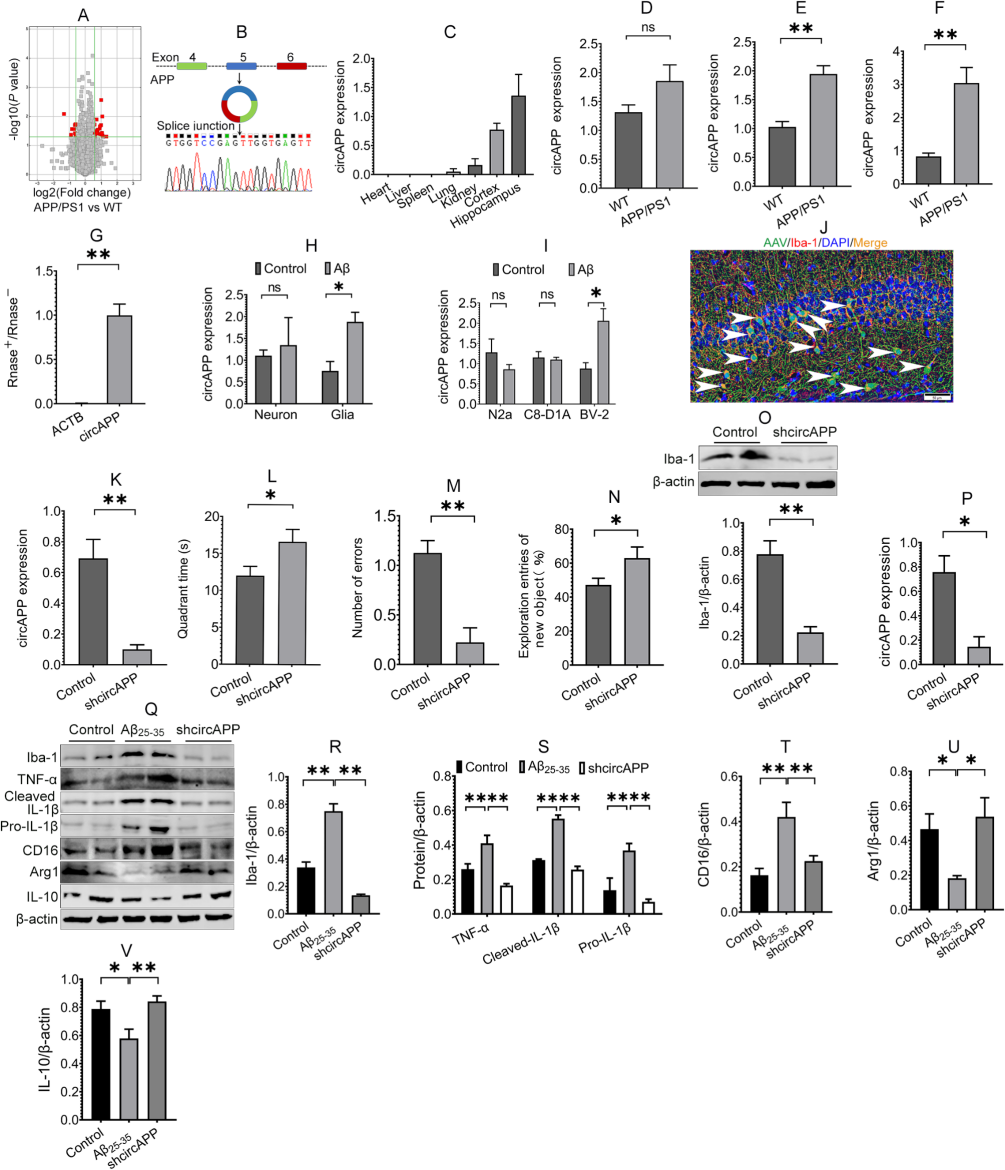
CircAPP is upregulated in microglia, and knockdown of circAPP in microglia improves cognitive function in APP/PS1 mice and modulates the microglial polarization in vitro. **(A)** A volcano plot showed dysregulated circRNAs in three hippocampal samples from APP/PS1 and WT mice identified via circRNA microarray, *n* = 3 samples/group. **(B)** Sanger sequencing of circAPP. **(C)** RT-PCR assay was performed to detect circAPP expression in the heart, liver, spleen, lung, kidney, cortex and hippocampus, *n* = 4. **(D ~ F)** RT-PCR assay was used to detect the expression of circAPP in 3- **(D**), 6- **(E)** and 10- **(F)** month-old APP/PS1 and age-matched WT mice, *n* = 4. (**G)** RNase R resistance of circAPP was assessed by RNase R treatment assay, *n* = 3. **(H)** The expression of circAPP in Aβ-treated primary neurons and glial cells was measured using RT-PCR assay, *n* = 3. **(I)** The expression of circAPP in Aβ-treated N2a, BV-2 and C8-D1A cells was detected using RT-PCR assay, *n* = 3 ~ 4. **(J)** The efficiency of infection of AAV2/9 containing a control shRNA with EGFP driven by microglia-specific promoters was evaluated using IF assay. AAVs (green) colocalized with a microglial marker, Iba-1 (red), in the hippocampus as indicated by white arrows. **(K)** RT-PCR assay was performed to measure circAPP expression in the hippocampus of mice after infection with shcircAPP AAVs for 6 weeks, *n* = 4. **(L ~ N)** The effect of circAPP knockdown on the cognitive function of APP/PS1 mice was evaluated using MWM test **(L)**, passive avoidance test **(M)** and novel object recognition task **(N)**, *n* = 8 ~ 10 mice. **(O)** The effect of circAPP knockdown on Iba-1 expression in the hippocampus of APP/PS1 mice was assessed using Western blot assay, *n* = 4. **(P)** CircAPP expression was detected using RT-PCR assay after BV-2 cells were transfected with shcircAPP LVs, *n* = 3. **(Q ~ V)** The effects of circAPP knockdown on the expression of Iba-1, TNF-α, pro- and cleaved IL-1β, CD16, Arg1 and IL-10 in Aβ-treated BV-2 cells were evaluated using Western blot assay, *n* = 3 ~ 4. All data in the figure are presented as mean ± SEM. Student’s *t* test **(D ~ I**,** K ~ P)** and one-way ANOVA **(R ~ V)** were used to assess statistically significant differences. *P < 0.05, **P < 0.01

To screen potential circRNAs in the early stage of AD, the hippocampal tissues of 6-month-old APP/PS1 and WT mice were collected, and circRNA microarray was performed. The results revealed that a total of 320 circRNAs were significantly dysregulated (*P* < 0.05) (Fig. 1A). We further screened these circRNAs according to their *P* values, fold changes, and conservation between humans and mice and found that circAPP (chr16:85056323–85082888) was significantly upregulated in the hippocampus of APP/PS1 and WT mice and was conserved between humans and mice via the Circbank online database. CircAPP is derived from exons 4, 5, and 6 of the *APP* gene. The PCR products of circAPP amplified by head-to-tail specific primers were analyzed by Sanger sequencing, and the results revealed that the sequence of the PCR products was completely in accordance with the circAPP sequence presented in circBase (Fig. 1B). We also detected the expression of circAPP in brain regions and different organs. The results revealed that circAPP was expressed primarily in the hippocampus, followed by the cortex and other organs, such as the kidney, lung, spleen, liver, and heart (Fig. 1C). Owing to the primary expression of circAPP in the hippocampus, we next detected the hippocampal expression of circAPP at different ages in APP/PS1 and WT mice. The results demonstrated that the hippocampal expression of circAPP started to increase at 3 months and was significantly upregulated after 6 months (Fig. 1D ~ F). These results suggest that circAPP may participate in AD pathogenesis.

### CircAPP is a stable circRNA that is upregulated in microglia after Aβ exposure

Next, we investigated the stability of circAPP. The RNase R resistance of circAPP was assessed using RNase R treatment assay. The results showed that circAPP was much more resistant to RNase R digestion than linear ACTB mRNA (Fig. 1G), suggesting the stability of circAPP. To better understand the role of circAPP in AD pathogenesis, we investigated the expression of circAPP in Aβ-treated primary neurons and glial cells. The results revealed that circAPP in Aβ-treated neurons exhibited a slightly upregulated trend with a fold change of 1.22, whereas circAPP was significantly upregulated in glial cells with a fold change of 2.49 after Aβ treatment (Fig. 1H). Furthermore, Aβ significantly upregulated circAPP in microglial BV-2 cells with a fold change of 2.35, whereas there was no significant difference in circAPP expression in Aβ-treated astrocytic C8-D1A and N2a cells with fold changes of 0.96 and 0.67, respectively (Fig. 1I), indicating that circAPP may be involved in Aβ-induced microglial activation.

### CircAPP knockdown in hippocampal microglia improves cognitive function of APP/PS1 mice

To investigate whether microglial circAPP affects behavioral deficits in AD, we first evaluated the efficiency of infection with AAV2/9 containing a control shRNA with EGFP driven by microglia-specific promoters. The results of IF assay showed that AAVs (green fluorescence) colocalized with the microglial marker Iba-1 (red fluorescence) in the hippocampus of mice after infection with AAVs for 3 weeks, suggesting the infection of AAVs in the hippocampal microglia (Fig. 1J). Next, we downregulated circAPP in hippocampal microglia of 6-month-old APP/PS1 mice by bilateral injection of AAV2/9-expressing control shRNA (control) or circAPP shRNA (shcircAPP) driven by microglia-specific promoters for 6 weeks. The results of the RT-PCR assay revealed that, compared with the control group, the hippocampal level of circAPP in the shcircAPP group was significantly reduced (Fig. 1K), indicating that circAPP is knocked down in hippocampal microglia.

Next, we assessed the impact of circAPP knockdown on cognitive function in APP/PS1 mice using the MWM test, passive avoidance test and novel object recognition task. The result of the MWM test revealed that circAPP knockdown significantly prolonged quadrant time of APP/PS1 mice (Fig. 1L), indicating an improvement in the spatial memory of circAPP knockdown. The result of the passive avoidance test demonstrated that memory errors were markedly decreased in the shcircAPP group compared with the control group (Fig. 1M), suggesting that circAPP knockdown ameliorates retention memory in APP/PS1 mice. The results of the novel object recognition task revealed that the percentage of exploring entries on novel object in the shcircAPP group was significantly increased compared with the control group (Fig. 1N), indicating that circAPP knockdown improves recognition memory in APP/PS1 mice. Taken together, these data suggest that circAPP knockdown in microglia improves cognitive function of APP/PS1 mice.

### CircAPP knockdown modulates microglial polarization and mitigates pathology in AD

**Fig. 2:**
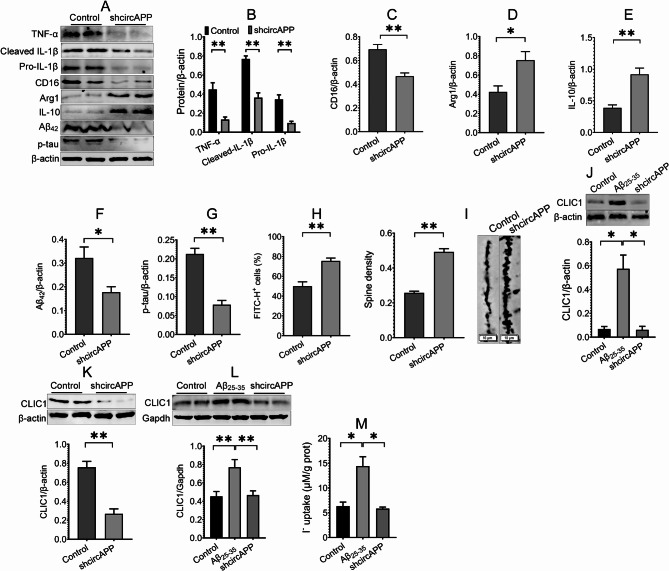
CircAPP knockdown in microglia modulates microglial polarization, improves AD pathology and inhibits CLIC1 expression and channel activity. **(A ~ G)** The effects of circAPP knockdown on the expression of TNF-α, Pro- and cleaved IL-1β, CD16, Arg1, IL-10, Aβ_42_ and p-tau in the hippocampus of APP/PS1 mice were evaluated using Western blot assay, *n* = 4. **(H)** The effect of circAPP knockdown on Aβ phagocytosis of BV-2 cells was assessed using flow cytometry assay, *n* = 3. **(I)** Golgi staining assay was performed to assess the effect of circAPP knockdown on spine density in the hippocampus of APP/PS1 mice, *n* = 3 mice. **(J ~ L)** The effects of circAPP knockdown on total CLIC1 expression in Aβ-treated BV-2 cells **(J**) and the hippocampus of APP/PS1 mice **(K)** and CLIC1 membrane expression in Aβ-treated BV-2 cells **(L)** were evaluated using Western blot assay, *n* = 4. **(M)** The effect of circAPP knockdown on CLIC1 channel activity in Aβ-treated BV-2 cells was assessed using functional chloride channel assay, *n* = 6. All data in the figure are presented as mean ± SEM. Student’s *t* test **(B ~ I**,** K**) and one-way ANOVA **(J**,** L**,** M**) were used to assess statistically significant differences. *P < 0.05, **P < 0.01

After behavioral tests, the influences of circAPP on microglial polarization and AD pathology were investigated. The results demonstrated that circAPP knockdown inhibited the expression of Iba-1 in Aβ-treated BV-2 cells and the hippocampus of APP/PS1 mice (Fig. 1O,Q and R), indicating that circAPP knockdown attenuates microglial activation. Next, we investigated whether circAPP knockdown influences microglial polarization. The results illustrated that circAPP knockdown downregulated the expression of the M1 marker CD16 and proinflammatory cytokines, including TNF-α, pro- and cleaved IL-1β, but upregulated the expression of the M2 marker Arg1 and the anti-inflammatory cytokine IL-10 in Aβ-treated BV-2 cells and the hippocampus of APP/PS1 mice (Fig. 1Q, S ~ V; Fig. 2A ~ E), indicating a modulatory role of circAPP in AD microglial polarization.

The microglial phenotype is closely associated with the ability of microglia to phagocytose Aβ [9]. Therefore, we determined the role of circAPP in Aβ phagocytosis using flow cytometry assay in which the population of FITC-positive cells was detected after BV-2 cells were treated with FITC-labeled Aβ_42_. The results revealed that the percentage of FITC-positive cells in the shcircAPP group was significantly increased compared with the control group (Fig. 2H), suggesting that Aβ phagocytosis is increased in circAPP-knockdown cells.

Abnormal microglial polarization is associated with increased levels of Aβ production and tau phosphorylation, leading to the impairment of synaptic morphology and cognitive deficits in AD [7, 9]. Therefore, we assessed the roles of circAPP in Aβ and phosphorylated tau (p-tau) expression and synaptic morphology. The results showed that circAPP knockdown significantly decreased the expression of Aβ_42_ and p-tau and increased spine density in the hippocampus of APP/PS1 mice (Fig. 2F, G and I), suggesting that circAPP knockdown in microglia mitigates AD pathology.

### CLIC1 is a potential target of circAPP

To elucidate the molecular mechanism of circAPP, a label-free proteomic was used to identify downstream targets in the hippocampus of WT mice overexpressing circAPP. A total of 142 proteins were dysregulated between the control and circAPP-overexpressing groups. Ten proteins with fold changes > 5.0 were further screened according to their sequence coverage. We found that CLIC1 had the highest sequence coverage (Supplementary Table S2) and has been reported to regulate the function of microglia treated with Aβ [33, 34]. Western blot analysis revealed that circAPP knockdown markedly decreased CLIC1 expression in Aβ-treated BV-2 cells and the hippocampus of APP/PS1 mice (Fig. 2J and K). We then detected the roles of circAPP in the expression of membrane CLIC1 and CLIC1 channel activity via Western blot and functional chloride channel assays, respectively, in Aβ-treated BV-2 cells. The results showed that Aβ induced increased membrane CLIC1 expression and channel activity, which could be reversed by circAPP knockdown (Fig. 2L and M). Together, these results indicate that CLIC1 is a potential target of circAPP.

### CLIC1 knockdown in microglia regulates microglial polarization and ameliorates pathology and cognitive function in AD

**Fig. 3:**
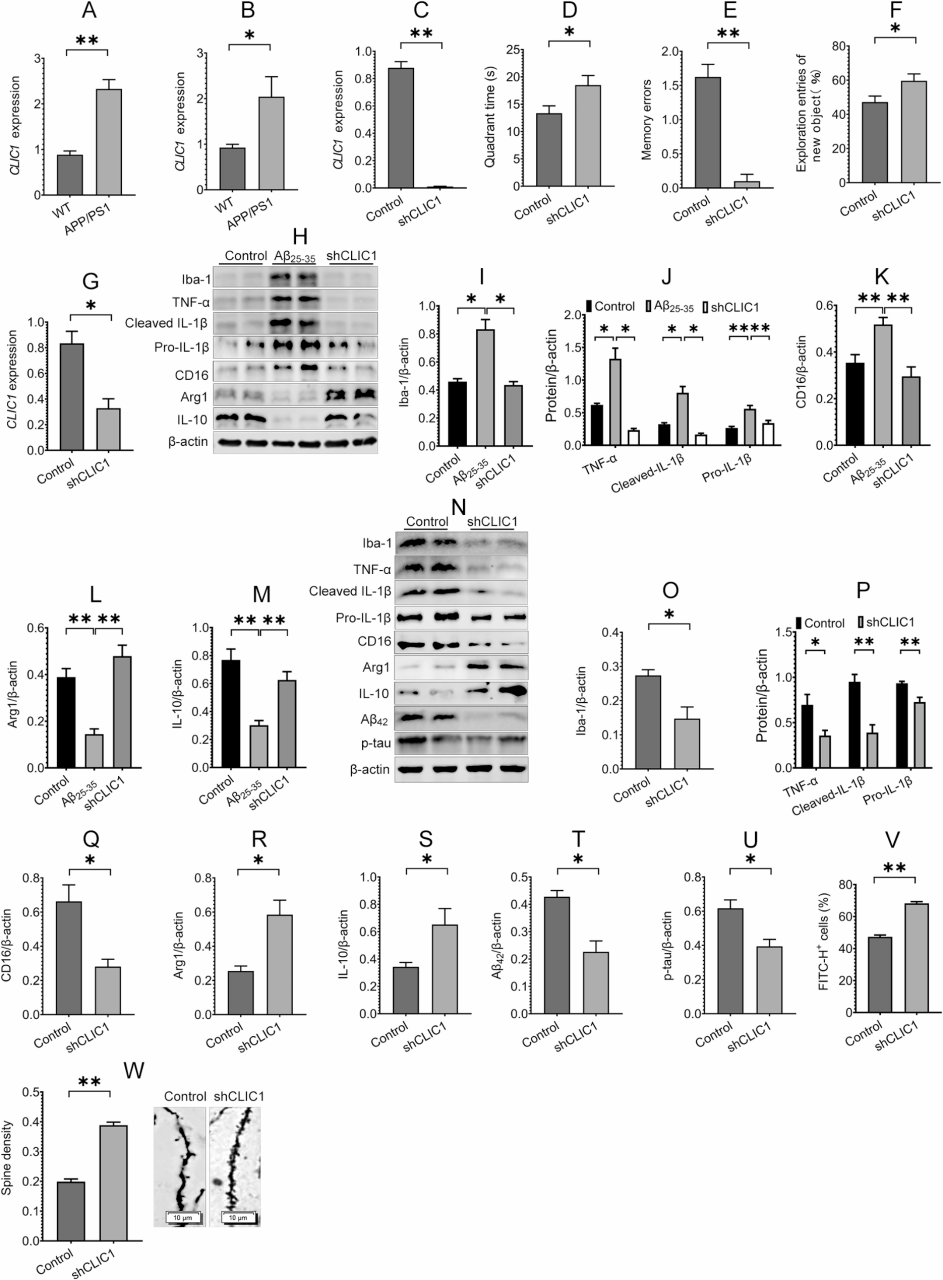
CLIC1 knockdown in microglia ameliorates cognitive function, regulates microglial polarization and ameliorates AD pathology. **(A**,** B)** CLIC1 expression in the hippocampus of 6-month-old **(A**) and 10-month-old **(B**) APP/PS1 and age-matched WT mice was detected using RT-PCR assay, *n* = 4. **(C)** RT-PCR assay was conducted to detect the expression of CLIC1 in the hippocampus of mice after hippocampal microglia were infected with shCLIC1 AAVs for 6 weeks, *n* = 4. **(D ~ F)** The effect of CLIC1 knockdown on the cognitive function of APP/PS1 mice was evaluated using MWM test **(D**), passive avoidance test **(E**) and novel object recognition task **(F**), *n* = 8 ~ 10 mice. **(G)** CLIC1 expression was detected using RT-PCR assay after BV-2 cells were transfected with shCLIC1 plasmids, *n* = 3. **(H ~ M)** The effects of CLIC1 knockdown on the expression of Iba-1, TNF-α, Pro- and cleaved IL-1β, CD16, Arg1 and IL-10 in Aβ-treated BV-2 cells were assessed using Western blot assay, *n* = 4. **(N ~ U)** The effects of CLIC1 knockdown on the expression of Iba-1, TNF-α, Pro- and cleaved IL-1β, CD16, Arg1, IL-10, Aβ_42_ and p-tau in the hippocampus of APP/PS1 mice were evaluated using Western blot assay, *n* = 3 ~ 4. **(V)** The effect of CLIC1 knockdown on Aβ phagocytosis of BV-2 cells was assessed using flow cytometry assay, *n* = 3 ~ 4. **(W)** Golgi staining assay was performed to assess the effect of CLIC1 knockdown on spine density in the hippocampus of APP/PS1 mice, *n* = 3 mice. All data in the figure are presented as mean ± SEM. Student’s *t* test (**A ~ G**,** O ~ W**) and one-way ANOVA (**I ~ M**) were used to assess statistically significant differences. *P < 0.05, **P < 0.01

CLIC1 is reportedly involved in Aβ phagocytosis and regulates Aβ-induced TNF-α release in microglia [34, 35], indicating the regulatory role of CLIC1 in microglial function. However, the role of CLIC1 in AD pathogenesis requires further investigation. We first detected the expression of CLIC1 in the hippocampus of APP/PS1 and WT mice at different ages and found that CLIC1 expression was significantly increased in the hippocampus of 6- and 10-month-old APP/PS1 mice compared with age-matched WT mice (Fig. 3A and B).

To investigate the role of CLIC1 in AD, we microinjected AAV2/9 expressing either shRNA control or shRNA CLIC1 (shCLIC1) driven by microglia-specific promoters in the hippocampus of APP/PS1 mice for 6 weeks. The results of the RT-PCR assay revealed that the expression of CLIC1 in the shCLIC1 group was significantly downregulated compared with the control group (Fig. 3C), indicating effective infection with AAVs. Importantly, CLIC1 knockdown in hippocampal microglia improved the cognitive function of APP/PS1 mice, as determined by the MWM test, passive avoidance test, and novel object recognition task (Fig. 3D ~ F). Additionally, CLIC1 knockdown caused a decrease in the expression of Iba-1, CD16, TNF-α, pro- and cleaved IL-1β, and an increase in the expression of Arg1 and IL-10 in Aβ-treated BV-2 cells and the hippocampus of APP/PS1 mice (Fig. 3H ~ S), suggesting that CLIC1 knockdown regulates microglial M1/M2 polarization in AD. Next, the roles of CLIC1 in microglial Aβ phagocytosis, the expression of Aβ and p-tau, and synaptic morphology were determined. The results revealed that CLIC1 knockdown increased Aβ phagocytosis of BV-2 cells, decreased Aβ and p-tau expression, and improved spine density in the hippocampus of APP/PS1 mice (Fig. 3T ~ W), suggesting an ameliorative role of CLIC1 knockdown in Aβ phagocytosis and AD pathology. Collectively, the above results indicate that CLIC1 knockdown in microglia regulates microglial polarization and alleviates pathology and cognitive function in AD.

### CircAPP regulates CLIC1 expression and channel activity through miR-1906

**Fig. 4:**
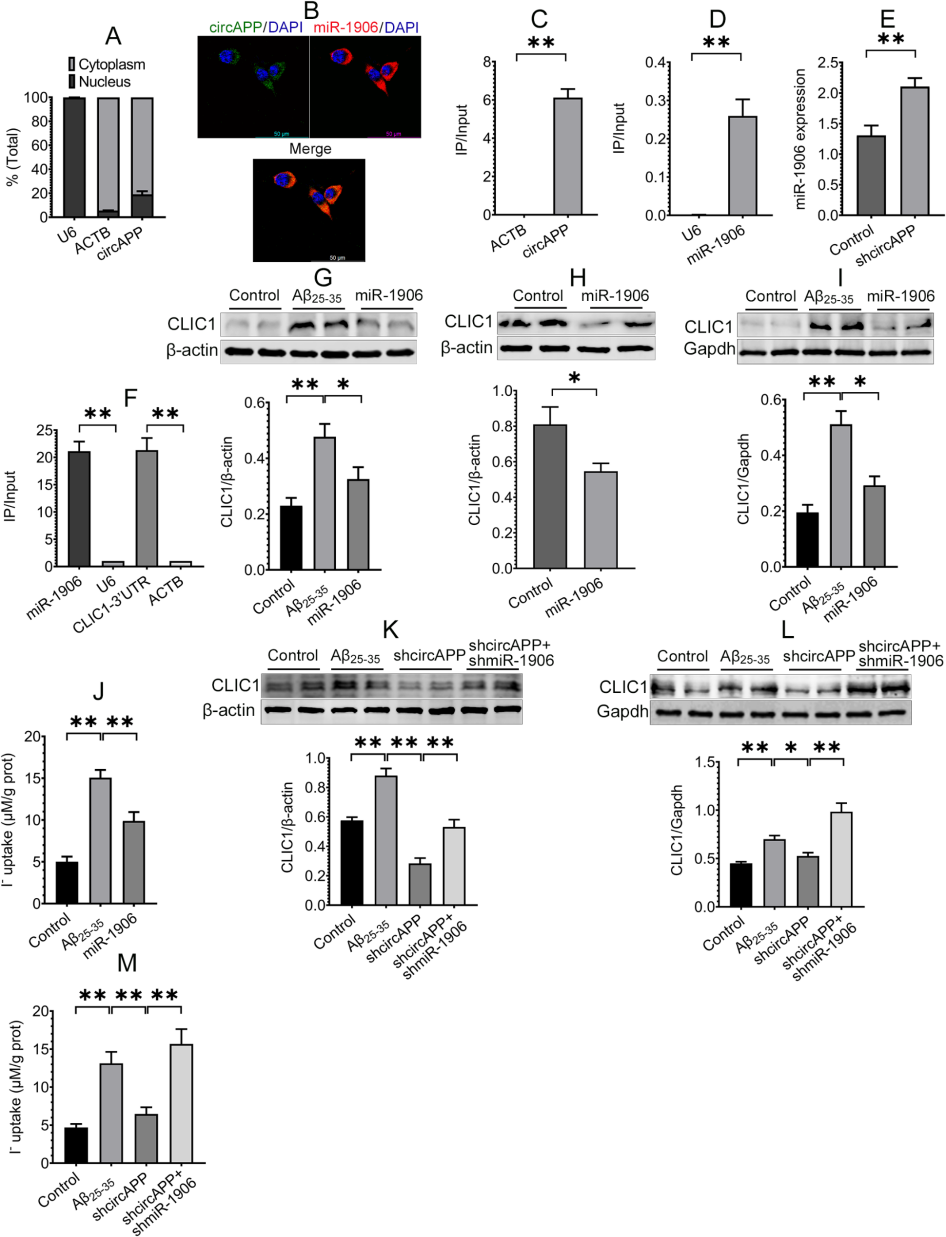
CircAPP regulates CLIC1 expression and channel activity through miR-1906. **(A)** RT-PCR assay was performed to detect circAPP expression in the cytoplasm and nucleus of BV-2 cells, *n* = 3. **(B)** The subcellular location of circAPP in BV-2 cells was detected using RNA-FISH assay. **(C**,** D)** The interaction between circAPP and miR-1906 was assessed using RNA pull-down assay, *n* = 4. **(E)** The effect of circAPP knockdown in microglia on miR-1906 expression in the hippocampus of APP/PS1 mice was evaluated using RT-PCR assay, *n* = 4. **(F)** The interaction between miR-1906 and 3’UTR of CLIC1 mRNA was assessed using RNA pull-down assay, *n* = 3. **(G ~ I)** The effects of miR-1906 overexpression on CLIC1 total expression in Aβ-treated BV-2 cells **(G)** and the hippocampus of APP/PS1 mice **(H)** and membrane expression in Aβ-treated BV-2 cells **(I)** were evaluated using Western blot assay, *n* = 4. **(J)** The effect of miR-1906 overexpression on CLIC1 channel activity in Aβ-treated BV-2 cells was assessed using functional chloride channel assay, *n* = 6. **(K**,** L)** The effects of miR-1906 overexpression on total **(K)** and membrane **(L)** expression of CLIC1 regulated by circAPP knockdown in Aβ-treated BV-2 cells were assessed using Western blot assay, *n* = 4. **(M)** The effect of miR-1906 overexpression on CLIC1 channel activity regulated by circAPP knockdown in Aβ-treated BV-2 cells was assessed using functional chloride channel assay, *n* = 6. All data in the figure are presented as mean ± SEM. Student’s *t* test **(C ~ F**,** H)** and one-way ANOVA **(G**,** I ~ M)** were used to assess statistically significant differences. *P < 0.05, **P < 0.01

Then, we sought to determine how circAPP regulates CLIC1 expression. We first detected the subcellular location of circAPP in BV-2 cells using RT-PCR and RNA-FISH assays. The results showed that circAPP was distributed primarily in the cytoplasm (Fig. 4A and B). Cytoplasmic circRNAs can modulate the expression of downstream targets by interacting with miRNAs [11]. Thus, we assumed that circAPP might regulate CLIC1 expression through miRNAs. We predicted miRNAs that may interact with both circAPP and CLIC1 via the miRanda, RNAhybrid, TargetScan and miRWalk online databases and found that miR-1906 was overlapped in the databases, indicating that miR-1906 may interact with circAPP and CLIC1. A biotin-labeled circAPP probe was subsequently designed to determine whether miR-1906 could be captured via RNA pull-down assay. The results revealed that both circAPP and miR-1906 are enriched, but not the negative controls ACTB and U6 (Fig. 4C and D). RNA-FISH assay unveiled that circAPP and miR-1906 were colocalized mainly in the cytoplasm (Fig. 4B). It has been reported that the interaction of circRNAs with miRNAs regulates miRNA expression [36, 37]. The influence of circAPP on miR-1906 expression was therefore investigated. The results revealed that circAPP knockdown in hippocampal microglia increased the miR-1906 level in the hippocampus of APP/PS1 mice (Fig. 4E). These results suggest that circAPP could interact with miR-1906 and inhibit its expression.

Accordingly, we determined whether miR-1906 interacted with CLIC1 and regulated CLIC1 expression. Because miRNAs regulate target expression by binding to 3’ untranslated regions (3’UTRs) of mRNAs [38], we designed a biotin-labeled miR-1906 probe to detect its interaction with the 3’UTR of CLIC1 mRNA via RNA pull-down assay and found the enrichment of miR-1906 and the 3’UTR of CLIC1 mRNA but not the negative controls (Fig. 4F), indicating the interaction of miR-1906 with CLIC1. We then detected whether miR-1906 affects CLIC1 expression and channel activity. The results demonstrated that miR-1906 overexpression decreased CLIC1 expression in Aβ-treated BV-2 cells and the hippocampus of APP/PS1 mice (Fig. 4G and H) and inhibited CLIC1 membrane protein expression and channel activity in Aβ-treated BV-2 cells (Fig. 4I and J). Next, we explored whether circAPP regulates CLIC1 expression and channel activity through miR-1906. The results revealed that the inhibitory effects of circAPP knockdown on total and membrane protein expression of CLIC1, as well as CLIC1 channel activity, were reversed by miR-1906 knockdown in Aβ-treated BV-2 cells (Fig. 4K ~ M). Taken together, these results indicate that the regulatory role of circAPP in CLIC1 is via miR-1906.

### miR-1906 overexpression in microglia modulates microglial polarization and improves pathology and cognitive function in AD

**Fig. 5:**
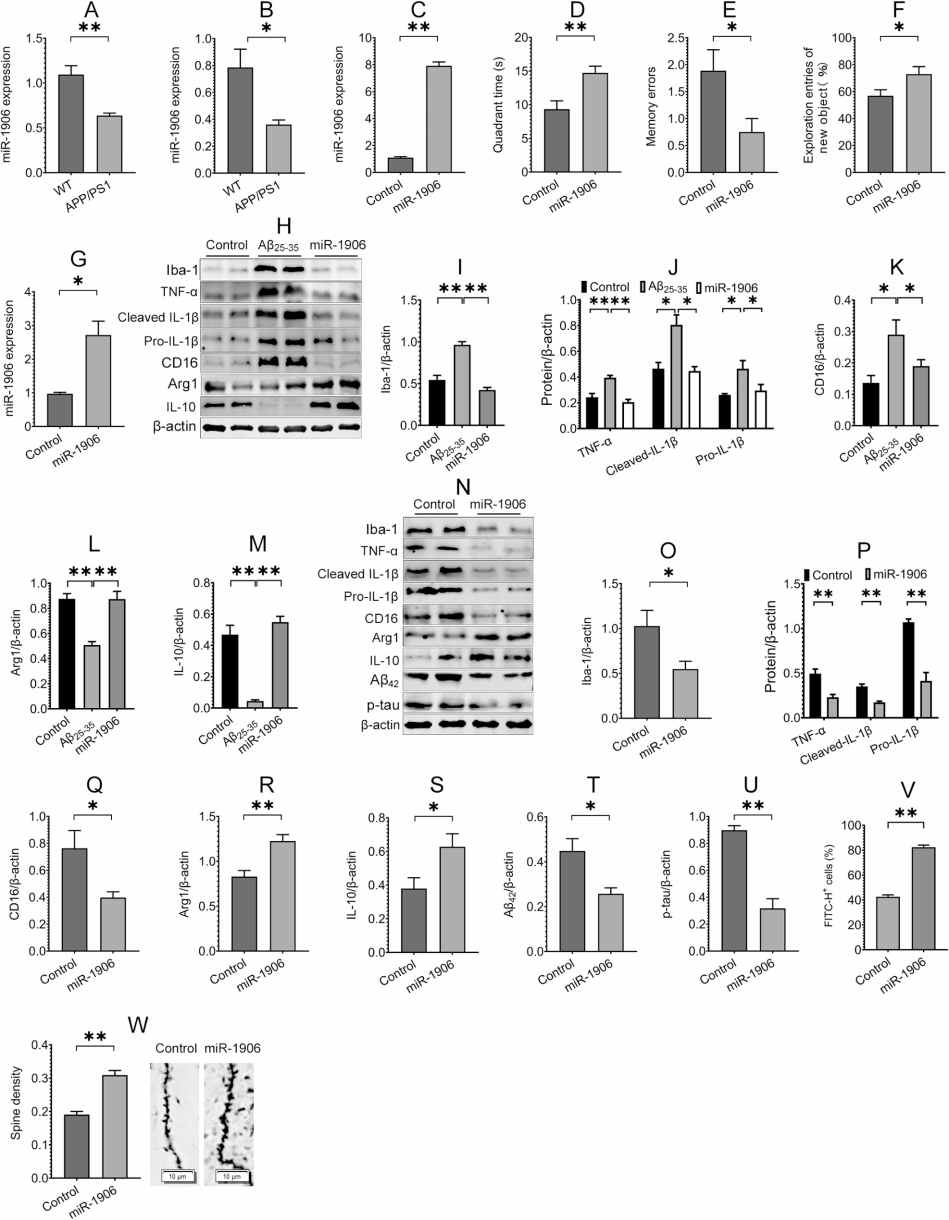
miR-1906 overexpression in microglia ameliorates cognitive function, modulates microglial polarization and improves AD pathology. **(A**,** B)** miR-1906 expression in the hippocampus of 6-month-old **(A**) and 10-month-old **(B**) APP/PS1 and age-matched WT mice was detected using RT-PCR assay, *n* = 3 ~ 4. **(C)** RT-PCR assay was conducted to detect miR-1906 expression in the hippocampus of mice after hippocampal microglia were infected with miR-1906 AAVs for 6 weeks, *n* = 3 ~ 4. **(D ~ F)** The effect of miR-1906 overexpression on the cognitive function of APP/PS1 mice was evaluated using MWM test **(D**), passive avoidance test **(E**) and novel object recognition task **(F)**, *n* = 7 ~ 9 mice. **(G)** The expression of miR-1906 was detected using RT-PCR assay after BV-2 cells were transfected with miR-1906 plasmids, *n* = 3 ~ 4. **(H ~ M)** The effects of miR-1906 overexpression on the expression of Iba-1, TNF-α, Pro- and cleaved IL-1β, CD16, Arg1 and IL-10 in Aβ-treated BV-2 cells were assessed using Western blot assay, *n* = 3 ~ 4. **(N ~ U)** The effects of miR-1906 overexpression on the expression of Iba-1, TNF-α, Pro- and cleaved IL-1β, CD16, Arg1, IL-10, Aβ_42_ and p-tau in the hippocampus of APP/PS1 mice were evaluated using Western blot assay, *n* = 4. **(V)** The effect of miR-1906 overexpression on Aβ phagocytosis of BV-2 cells was assessed using flow cytometry, *n* = 4. **(W)** Golgi staining assay was performed to assess the effect of miR-1906 overexpression on spine density in the hippocampus of APP/PS1 mice, *n* = 3 mice. All data in the figure are presented as mean ± SEM. Student’s *t* test **(A ~ G**,** O ~ W**) and one-way ANOVA **(I ~ M)** were used to assess statistically significant differences. *P < 0.05, **P < 0.01

miR-1906 has been reported to inhibit the expression of proinflammatory cytokines in the peri-infarct area and ischemic core of mice with ischemic stroke [39], indicating a regulatory role of miR-1906 in neuroinflammation. Because the role of miR-1906 in AD pathogenesis is still unclear, we first detected the expression of miR-1906 in the hippocampus of APP/PS1 and WT mice at different ages. The results illustrated that miR-1906 expression was significantly decreased in the hippocampus of 6- and 10-month-old APP/PS1mice compared with age-matched WT mice (Fig. 5A and B).

The findings that miR-1906 regulates neuroinflammation [39] and is downregulated in AD mice prompted us to explore whether miR-1906 may play a regulatory role in the microglial phenotype in AD. We injected AAV2/9 preparations carrying control or miR-1906 driven by microglia-specific promoters into the hippocampus of APP/PS1 mice for 6 weeks. The results of the RT-PCR assay revealed a significant increase in miR-1906 expression in the miR-1906-overexpressing group compared with the control group (Fig. 5C), suggesting effective infection with AAVs. Notably, miR-1906 overexpression in hippocampal microglia improved the cognitive function of APP/PS1 mice, as detected by the MWM test, passive avoidance test, and novel object recognition task (Fig. 5D ~ F). Furthermore, miR-1906 overexpression attenuated the expression of Iba-1, CD16, TNF-α, pro- and cleaved IL-1β, and promoted the expression of Arg1 and IL-10 in Aβ-treated BV-2 cells and the hippocampus of APP/PS1 mice (Fig. 5H ~ S), suggesting that miR-1906 overexpression modulates microglial M1/M2 polarization in AD. We subsequently determined the roles of miR-1906 in microglial Aβ phagocytosis, Aβ and p-tau expression, and synaptic morphology. The results showed that miR-1906 overexpression promoted Aβ phagocytosis of BV-2 cells, downregulated the expression of Aβ and p-tau, and improved spine density in the hippocampus of APP/PS1 mice (Fig. 5T ~ W), suggesting a role of miR-1906 in the amelioration of Aβ phagocytosis and AD pathology. Together, these results indicate that miR-1906 overexpression in microglia modulates microglial polarization and improves pathology and cognitive function in AD.

**CircAPP regulates microglial polarization via miR-1906/CLIC1 axis **in vitro

**Fig. 6:**
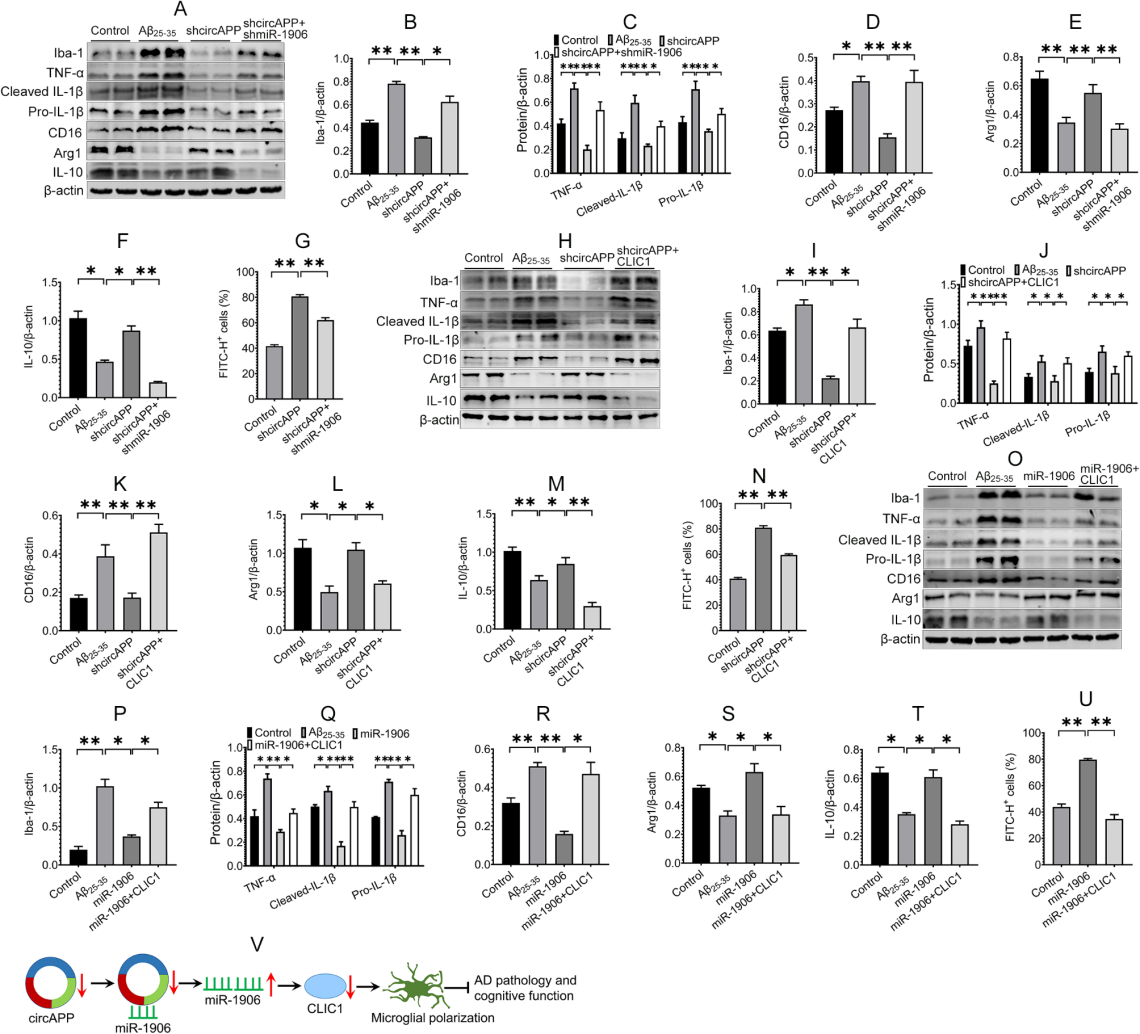
CircAPP regulates microglial polarization through miR-1906/CLIC1 axis in vitro.**(A ~ F)** The effects of miR-1906 knockdown on the expression of Iba-1, TNF-α, Pro- and cleaved IL-1β, CD16, Arg1 and IL-10 regulated by circAPP knockdown in Aβ-treated BV-2 cells were assessed via Western blot assay, *n* = 4. **(G)** The effect of miR-1906 knockdown on Aβ phagocytosis of BV-2 cells regulated by circAPP knockdown was assessed using flow cytometer assay, *n* = 3 ~ 4. **(H ~ M)** The effects of CLIC1 overexpression on the expression of Iba-1, TNF-α, Pro- and cleaved IL-1β, CD16, Arg1 and IL-10 regulated by circAPP knockdown in Aβ-treated BV-2 cells were assessed using Western blot assay, *n* = 4. **(N)** The effect of CLIC1 overexpression on Aβ phagocytosis of BV-2 cells regulated by circAPP knockdown was assessed using flow cytometry assay, *n* = 4 ~ 5. **(O ~ T)** The effects of CLIC1 overexpression on the expression of Iba-1, TNF-α, Pro- and cleaved IL-1β, CD16, Arg1 and IL-10 regulated by miR-1906 overexpression in Aβ-treated BV-2 cells were assessed using Western blot assay, *n* = 4. **(U)** The effect of CLIC1 overexpression on Aβ phagocytosis of BV-2 cells regulated by miR-1906 overexpression was assessed using flow cytometry assay, *n* = 3 ~ 4. **(V)** Schematic representation of proposed mechanism of circAPP in AD microglial polarization, pathology and cognitive function. CircAPP was upregulated in Aβ-treated microglial cells and the hippocampus of APP/PS1 mice. CircAPP knockdown inhibited its interaction with miR-1906 and increased miR-1906 expression, which in turn retarded CLIC1 expression and channel activity, thereby regulating microglial polarization and ameliorating AD pathology and cognitive function. All data in the figure are presented as mean ± SEM. One-way ANOVA was used to assess statistically significant differences. *P < 0.05, **P < 0.01

We subsequently investigated whether circAPP regulates the microglial phenotype via miR-1906 and CLIC1. The results revealed that circAPP knockdown downregulated the expression of Iba-1, CD16, TNF-α, pro- and cleaved IL-1β, upregulated the expression of Arg1 and IL-10 in Aβ-treated BV-2 cells and promoted Aβ phagocytosis in BV-2 cells (Fig. 6A ~ N), indicating a modulatory role of circAPP knockdown in microglial M1/M2 polarization. Notably, the regulatory role of circAPP knockdown in the microglial polarization could be reversed by miR1906 knockdown or CLIC1 overexpression (Fig. 6A ~ N). In addition, CLIC1 overexpression could inhibit the regulatory role of miR-1906 overexpression in microglial polarization (Fig. 6O ~ U). These results suggest that circAPP regulates microglial polarization via miR-1906/CLIC1 axis in vitro.

## Discussion

In the present study, we not only characterized a novel circRNA circAPP, that is upregulated in Aβ-treated microglia and the hippocampus of APP/PS1 mice but also investigated the regulatory roles of circAPP and its downstream mediators miR-1906 and CLIC1 in microglial polarization in vitro and in vivo, and AD pathology and cognitive function in vivo. Furthermore, we demonstrated that circAPP could regulate CLIC1 expression and channel activity by interacting with miR-1906 and affecting miR-1906 expression. Overall, the present study provides important evidence indicating that circAPP regulates AD microglial polarization via the miR-1906/CLIC1 axis, shedding light on the novel role of circAPP in AD.

Aberrant microglial polarization phenotype has been demonstrated to play a key role in AD progression [4, 7, 40]. M1 microglia activated by Aβ aggregates cause the sustained release of proinflammatory cytokines and reduce the phagocytosis of Aβ, whereas the M2 phenotype, which possesses anti-inflammatory properties and increases Aβ phagocytosis, is prohibited during AD progression [2, 4, 7]. The disequilibrium of microglial polarization causes neuroinflammation and Aβ generation, leading to impaired synaptic function and cognitive function in AD [4, 7, 40]. To date, the regulatory roles of circRNAs in the microglial phenotype have not been fully characterized. In this study, we found that circAPP began to increase in the hippocampus of 3-month-old APP/PS1 mice and was further elevated with age. Further results revealed that circAPP was upregulated in Aβ-treated microglia, raising the possibility that it might have a regulatory role in the microglial polarization. This possibility was validated in our further study, indicating the function of circAPP in regulating the expression of M1 and M2 markers in vitro and in vivo, and Aβ phagocytosis in vitro. Since neuroinflammation caused by abnormal microglial polarization is associated with AD pathology, including Aβ production, tau hyperphosphorylation and synaptic impairment, as well as the cognitive decline in AD [2, 4], we detected the regulatory roles of circAPP in the expression of Aβ and p-tau, synaptic morphology, and cognitive function. The results demonstrated that circAPP knockdown reduced the hippocampal levels of Aβ and p-tau and improved the hippocampal synaptic morphology and cognitive function of APP/PS1 mice. Overall, our study elucidated the regulatory roles of circAPP in microglial polarization and AD pathology and cognitive function. Up to now, the roles of circRNAs in brain cells during AD progression remain largely unknown. It would be necessary to identify circRNAs in brain cells during AD progression using single nucleus RNA sequencing technology and characterize their regulatory roles in AD in future studies.

In this study, we took advantage of proteomics to determine the downstream targets of circAPP and found that CLIC1 had a high possibility, which was confirmed by the results showing the regulatory roles of circAPP in CLIC1 expression and channel activity. It has been demonstrated that Aβ can increase CLIC1 expression and improve its membrane localization and channel activity in microglia [33]. Inhibition of CLIC1 expression or channel activity can increase Aβ phagocytosis and decrease proinflammatory cytokine release in Aβ-treated microglia [35]. On the basis of these findings, we speculated that CLIC1 might be involved in microglial polarization in AD. Our study revealed that CLIC1 expression was increased in the hippocampus of APP/PS1 mice. CLIC1 knockdown in microglia could regulate microglial M1/M2 polarization in vitro and in vivo, ameliorate AD pathology and cognitive function in vivo, indicating modulatory roles of CLIC1 in AD microglial polarization, pathology and cognitive function. However, whether the regulatory role of CLIC1 in microglial polarization is associated with channel activity and the mechanisms by which CLIC1 regulates microglial polarization and AD pathology should be investigated in future studies.

Multiple lines of evidence show that cytoplastic circRNAs can regulate target expression by interacting with miRNAs [11, 36, 37]. RT-PCR and RNA-FISH assays revealed that circAPP was located mainly in the cytoplasm, indicating that circAPP might interact with miRNAs to regulate CLIC1 expression. We predicted miRNAs potentially interacting with both circAPP and CLIC1 via online databases, and miR-1906 was identified as a candidate. The interaction of miR-1906 with circAPP was confirmed via RNA pull-down and RNA-FISH assays. It has been reported that the interaction of circRNA with miRNAs affects miRNA expression via regulating miRNA degradation [41]. We detected miR-1906 expression upon circAPP knockdown and found that circAPP knockdown could increase miR-1906 expression. However, whether circAPP affects miR-1906 degradation needs to be investigate in future studies. Additionally, the interaction of miR-1906 with CLIC1 was validated via RNA pull-down assay. Further results revealed that miR-1906 could decrease CLIC1 expression and channel activity, and the regulatory roles of circAPP knockdown in CLIC1 expression and channel activity could be reversed by miR-1906, indicating that circAPP can regulate CLIC1 expression and channel activity by interacting with miR-1906 and regulating miR-1906 expression.

It has been reported that miR-1906 has anti-inflammatory effects in mice with ischemic stroke [39]. However, its role in AD is unclear. The present study revealed that hippocampal miR-1906 expression was downregulated in 6- and 10-month-old APP/PS1 mice. miR-1906 overexpression could modulate microglial M1/M2 polarization in vitro and in vivo, and improve AD pathology and cognitive function in vivo, indicating regulatory roles of miR-1906 in AD microglial polarization, pathology and cognitive function. Further experiments revealed that the role of miR-1906 in microglial polarization could be inhibited by CLIC1 overexpression. Moreover, the role of circAPP knockdown in modulating microglial polarization could be prohibited by either miR1906 knockdown or CLIC1 overexpression, indicating that circAPP modulates microglial polarization through miR-1906/CLIC1 axis. Notably, although overexpression of miR-1906 or knockdown of CLIC1 largely phenocopied circAPP knockdown, it was not identical to the level provided by circAPP knockdown in AD microglial polarization, pathology and cognitive function, indicating that other potential targets might be involved in circAPP-mediated regulation of AD microglial polarization and pathology.

Several limitations have to be interpreted. First, an upregulation of circAPP was found in the hippocampus of APP/PS1 mice, which may result from exogenous mutant *APP*. The expression of circAPP in other AD mouse models should be detected in future studies. Second, a hippocampal injection technique was conducted in the study, viral vectors may influence other brain regions due to the circulation of cerebrospinal fluid. Additionally, microglia-specific promoters may exhibit off-target expression in other cell types expressing similar markers [42]. Therefore, the impact of circAPP, miR-1906 and CLIC1 in other brain regions or cell types should be considered. Third, we found a slightly upregulated trend but not significant upregulation in circAPP expression in Aβ-treated primary neurons, which might be due to the limited repeated experimental times. Further studies should be designed to detect circAPP expression in a larger sample size in vivo and investigate whether circAPP affects neuronal function in AD.

## Conclusions

Taken together, our results elucidate the regulatory role of circAPP in AD microglial polarization via miR-1906/CLIC1 axis, indicating that circAPP may play a critical role in AD pathogenesis. This study expands our understanding of the functions of cerebral circRNAs and offers new insights into the development of potential preventive strategies and effective therapies for AD.

## Electronic supplementary material

Below is the link to the electronic supplementary material.

Supplementary Material 1

Supplementary Material 2

Supplementary Material 3

## Data Availability

The data that support the findings of this study are available from the corresponding author upon reasonable request.

## Abbreviations

AD: Alzheimer’s disease
circRNAs: Circular RNAs
Aβ: Amyloidβ
p-tau: Phosphorylated tau
CD16: Cluster of differentiation 16
TNF-α: Tumor necrosis factor-α
IL-1β: Interleukin-1β
ARG1: Arginase1
IL-10: Interleukin-10
miRNA: microRNA
APP: Amyloid precursor protein
CLIC1: Chloride intracellular channel 1
WT: Wild-type
DMEM: Dulbecco’s modified Eagle’s medium
N2a: Neuro-2a
AAVs: Adeno-associated viruses
MWM: Morris water maze
FISH: RNA-fluorescence in situ hybridization
ANOVA: One-way analysis of variance
3’UTR: 3’ untranslated region
