# Supplementary material for: Circular RNA APP contributes to Alzheimer’s disease pathogenesis by modulating microglial polarization via miR-1906/CLIC1 axis
Source: Alzheimers Res Ther. 2025 Feb 14;17:44. doi: 10.1186/s13195-025-01698-7 (PMC11829462; doi:10.1186/s13195-025-01698-7)
Supplement: Supplementary file 1 — Supplementary Material 1 [file 13195_2025_1698_MOESM1_ESM.docx]

**Supplementary table 1: The sequences of circAPP and its primers, probes and oligonucleotides used in the study**

| **Primers for PCR (5’-3’)** | |
| --- | --- |
| circAPP | TTGGTGAGTTTGTGAGCGACGCCCTTCTCGTGCCCGACAAGTGCAAGTTCCTACACCAGGAGCGGATGGATGTTTGTGAGACCCATCTTCACTGGCACACCGTCGCCAAAGAGACATGCAGCGAGAAGAGCACTAACTTGCACGACTATGGCATGCTGCTGCCCTGCGGCATCGACAAGTTCCGAGGGGTAGAGTTTGTATGCTGCCCGTTGGCCGAGGAAAGCGACAGCGTGGATTCTGCGGATGCAGAGGAGGATGACTCTGATGTCTGGTGGGGTGGAGCGGACACAGACTACGCTGATGGCGGTGAAGACAAAGTAGTAGAAGTCGCCGAAGAGGAGGAAGTGGCTGATGTTGAGGAAGAGGAAGCTGATGATGATGAGGATGTGGAGGATGGGGACGAGGTGGAGGAGGAGGCCGAGGAGCCCTACGAAGAGGCCACCGAGAGAACAACCAGCACTGCCACCACCACCACAACCACCACTGAGTCCGTGGAGGAGGTGGTCCGAG |
| Divergent circAPP | F: GTGGAGGAGGTGGTCCGAGTTG  R: GCAGCAGCATGCCATAGTCGTG |
| miR-1906 | TATATATGCAGCAGCCTGAGGCA |
| CLIC1 | F: CGTGAAGGCTGGCAGTGATGG  R: AGTCTCTGTCCGTCTCTTGGTGTC |
| CLIC1 3’UTR | F: GGCTTCCACGTTGCTGCATAATG  R: GTTGTCCTACCCACCCCATCCC |
| ACTB | F: GTGCTATGTTGCTCTAGACTTCG  R: ATGCCACAGGATTCCATACC |
| U6 | Sangon Biotech |
| **FISH probes (5’-3’)** | |
| circAPP (FAM) | ACAAACTCACCAACTCGGACCACCTCCT |
| miR-1906 (Cy3) | AGCCCTGCCTCAGGCTGCTGCA |
| **Biotinylated probes (5’-3’)** | |
| circAPP | ACAAACTCACCAACTCGGACCACCTCCT |
| miR-1906 | AGCCCTGCCTCAGGCTGCTGCA |
| **Plasmids, lentiviruses and AAV shRNA** | |
| circAPP shRNA | TCCGAGTTGGTGAGTTTGT |
| CLIC1 shRNA | GGGAGTCACCTTCAACGTT |
| miR-1906 shRNA | AGCCCTGCCTCAGGCTGCTG |
