# Supplementary material for: Circular RNA APP contributes to Alzheimer’s disease pathogenesis by modulating microglial polarization via miR-1906/CLIC1 axis
Source: Alzheimers Res Ther. 2025 Feb 14;17:44. doi: 10.1186/s13195-025-01698-7 (PMC11829462; doi:10.1186/s13195-025-01698-7)
Supplement: Supplementary file 2 — Supplementary Material 2 [file 13195_2025_1698_MOESM2_ESM.docx]

**Supplementary table 2: The sequence coverage of proteins with fold change>5.0 and efficiency of infection of AAV overexpressing circAPP in the hippocampus of WT mice, and miR-1906 shRNA and CLIC1 overexpression plasmids in BV-2 cells**

| **Sequence coverage of proteins with fold change>5.0** | | |
| --- | --- | --- |
| Protein name | Sequence coverage (%) | Expression |
| Clic1 | 19.5 | Upregulated |
| Dync1li2 | 14.2 | Upregulated |
| Dazap1 | 11.6 | Upregulated |
| Qki | 11.2 | Upregulated |
| Cdh2 | 7.8 | Upregulated |
| Rabgap1 | 4.8 | Upregulated |
| Ktn1 | 2.4 | Upregulated |
| Itm2c | 8.6 | Downregulated |
| Kiaa1671 | 13.8 | Downregulated |
| Prdx4 | 12.7 | Downregulated |
| **Efficiency of infection of AAV overexpressing circAPP in the hippocampus of WT mice, and miR-1906 shRNA and CLIC1 overexpression plasmids in BV-2 cells** | | |
| 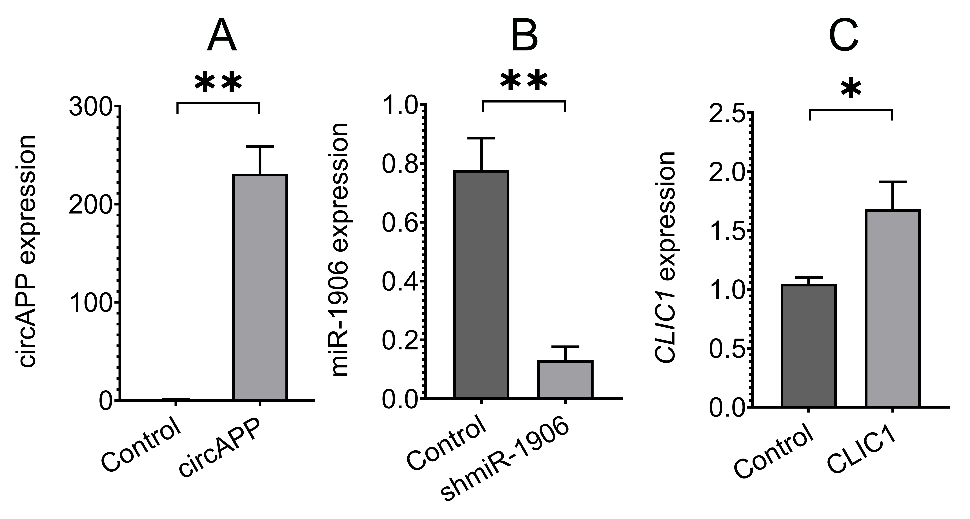 | | |
| **A** AAVs overexpressing circAPP were injected into the hippocampus of WT mice for 6 weeks. The hippocampus was dissected out and the expression of circAPP was assessed by RT-PCR assay, n=4. **B,C** BV-2 cells were transfected with miR-1906 shRNA and CLIC1 overexpression plasmids and the expression of miR-1906 and CLIC1 in BV-2 cells after transfection was detected by RT-PCR assay, n=4. The data of each group were presented as mean±SEM. The data were statistically analyzed using Student’s *t* test to compare the difference between two groups using SPSS software for Windows 20.0. **P*<0.05, ***P*<0.01. | | |
