# Supplementary material for: Circular RNA APP contributes to Alzheimer’s disease pathogenesis by modulating microglial polarization via miR-1906/CLIC1 axis
Source: Alzheimers Res Ther. 2025 Feb 14;17:44. doi: 10.1186/s13195-025-01698-7 (PMC11829462; doi:10.1186/s13195-025-01698-7)
Supplement: Supplementary file 3 — Supplementary Material 3 [file 13195_2025_1698_MOESM3_ESM.pdf]

Figure 1O

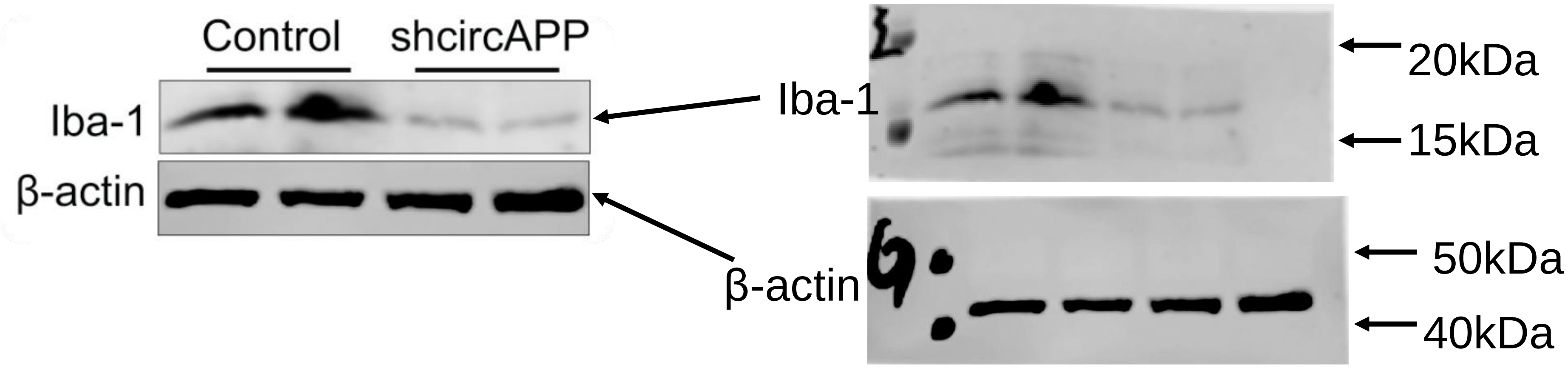
Control
shcircAPP
Iba-1
β-actin
Iba-1
β-actin
20kDa
15kDa
50kDa
40kDa

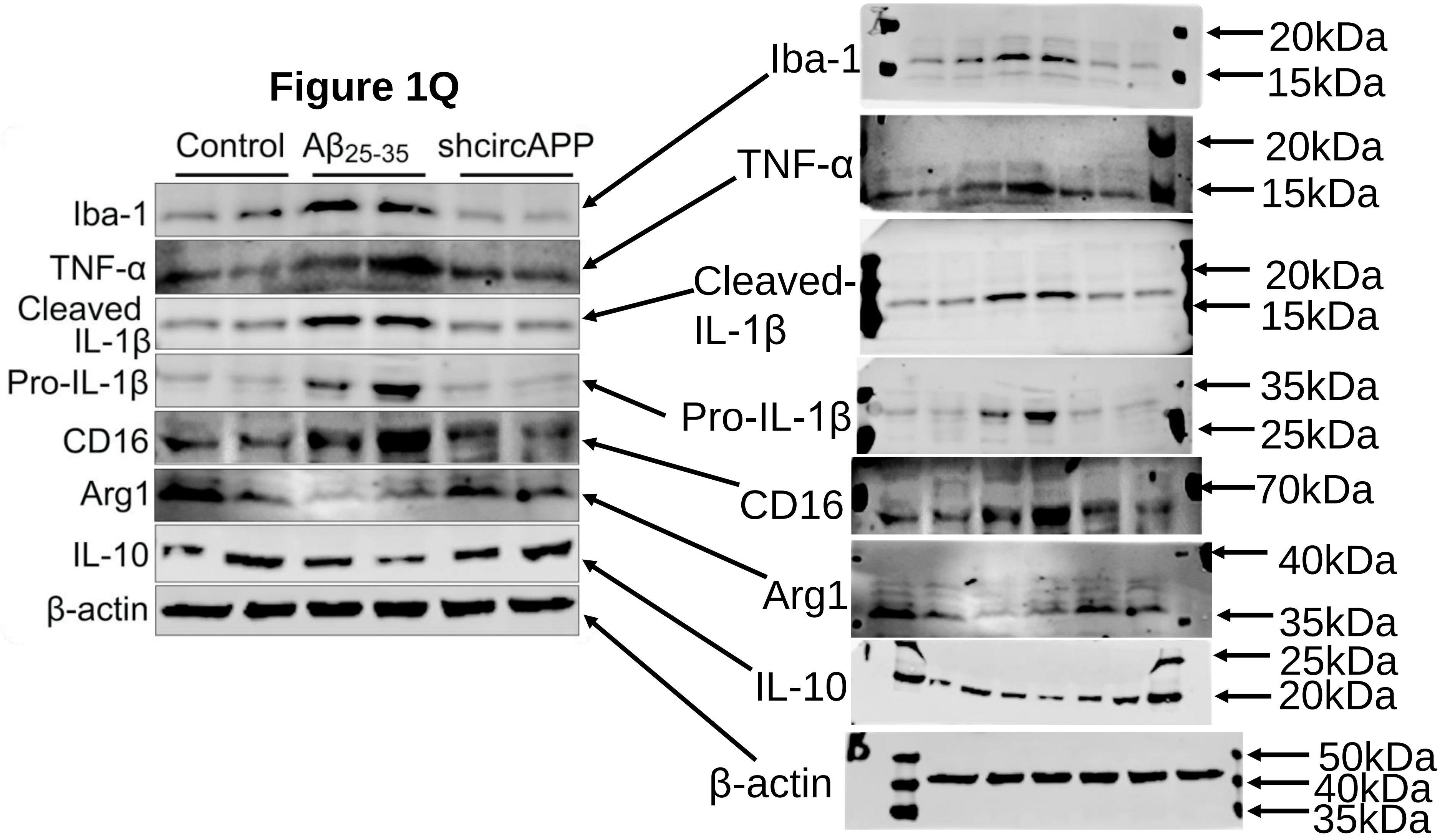
Figure 1Q
Control
Aβ25-35
shcircAPP
Iba-1
TNF-α
Cleaved IL-1β
Pro-IL-1β
CD16
Arg1
IL-10
β-actin
20kDa
15kDa
35kDa
25kDa
70kDa
40kDa
50kDa

Figure 2A

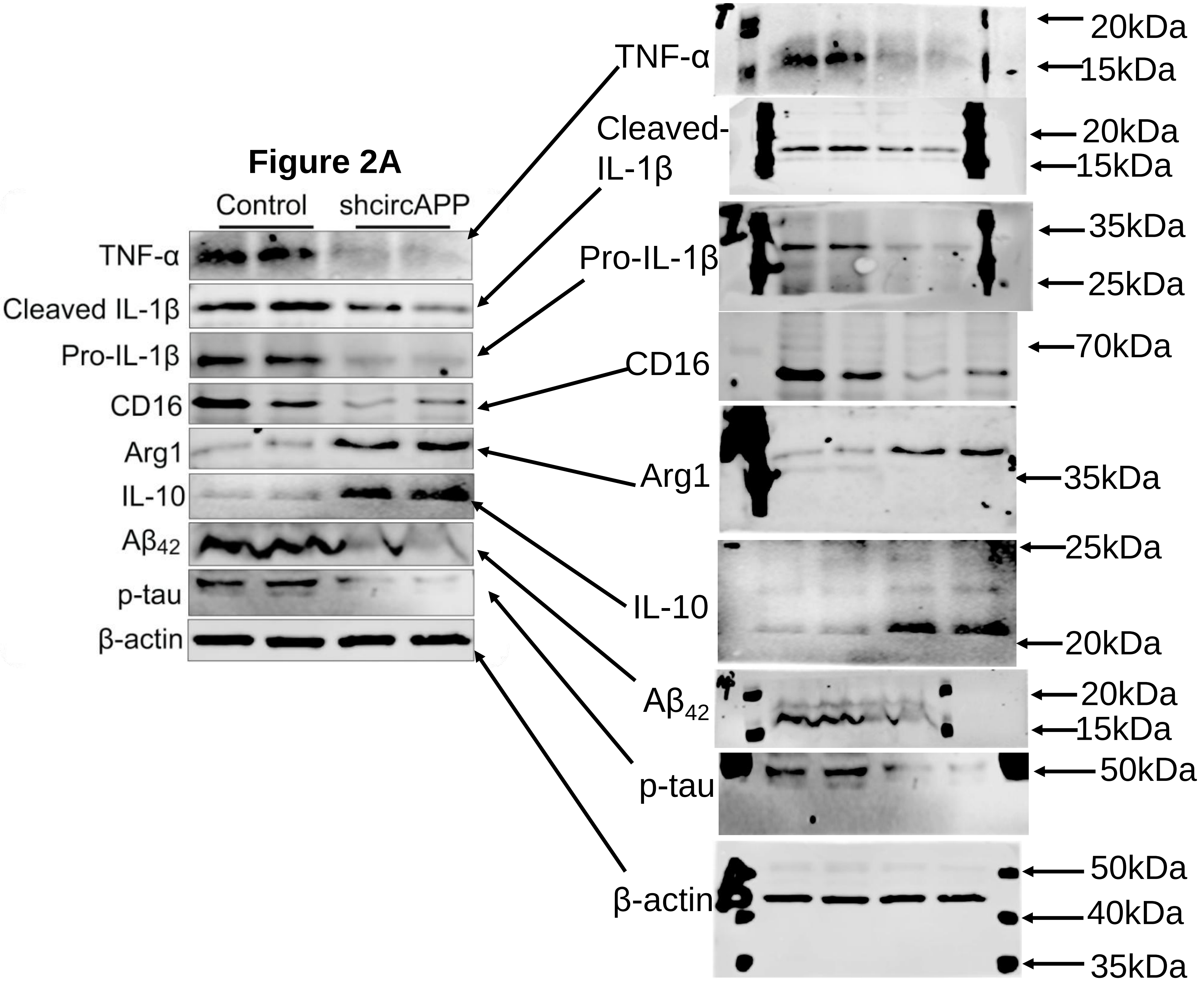
Control
shcircAPP
TNF-α
Cleaved IL-1β
Pro-IL-1β
CD16
Arg1
IL-10
Aβ42
p-tau
β-actin
20kDa
15kDa
Cleaved-IL-1β
35kDa
25kDa
70kDa
50kDa
40kDa

Figure 2J

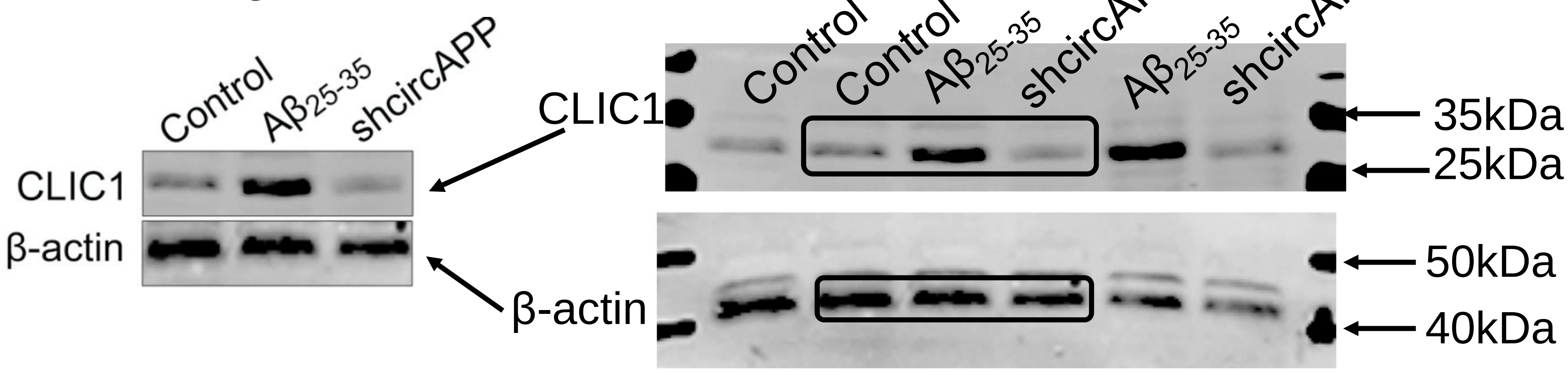

Figure 2K

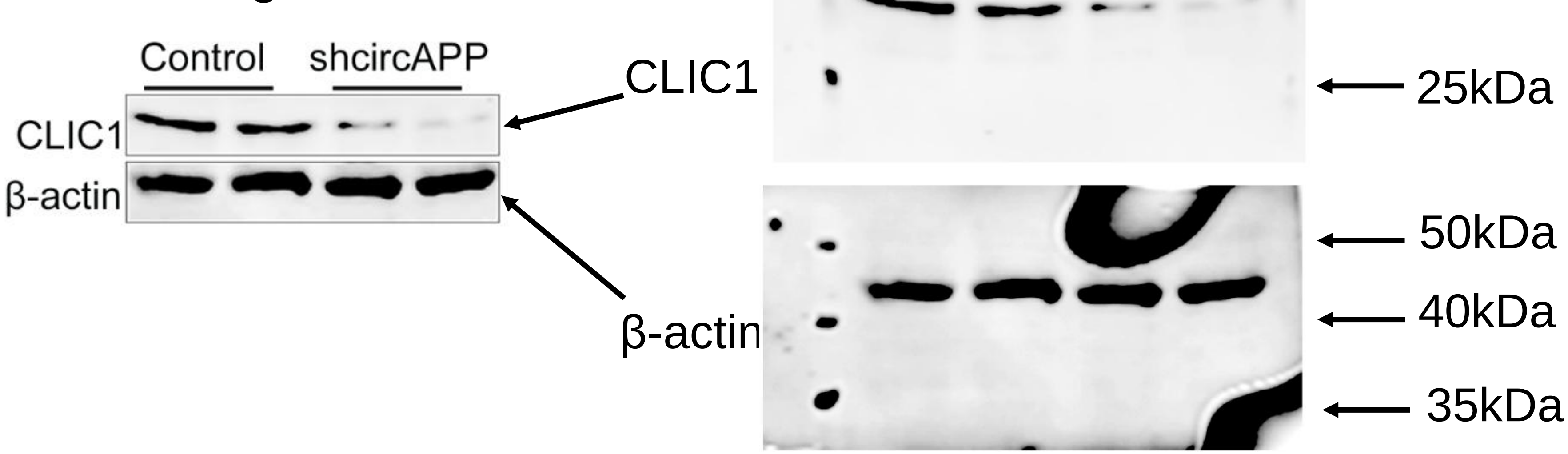

Figure 2L

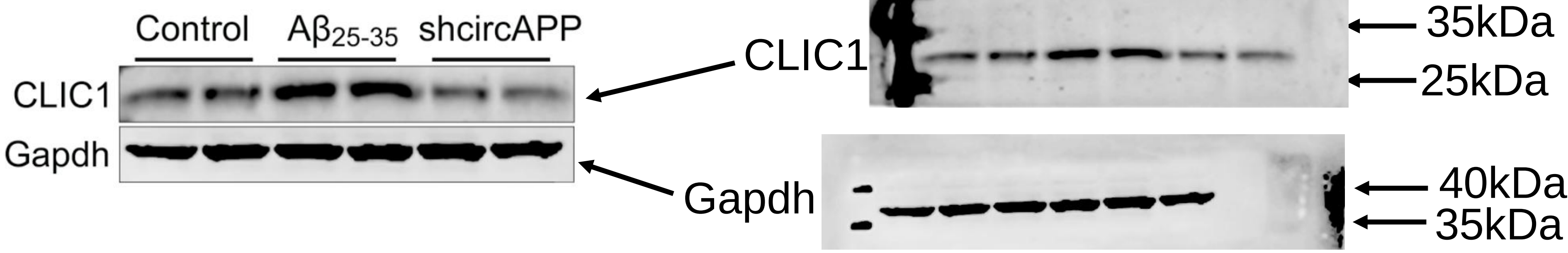
Control
Aβ25-35
shcircAPP
CLIC1
Gapdh
CLIC1
35kDa
25kDa
Gapdh
40kDa
35kDa

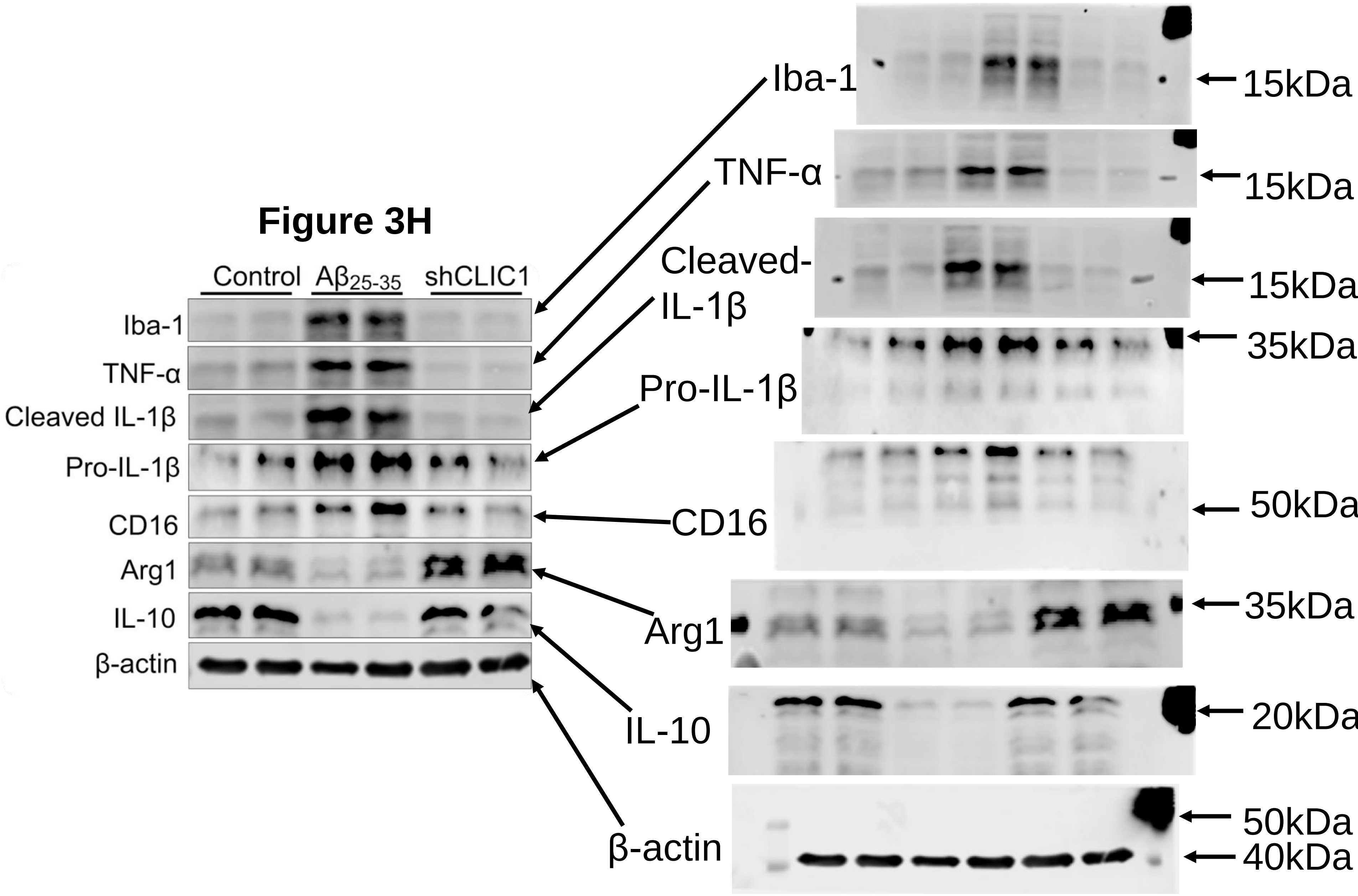
Figure 3H
Control
Aβ25-35
shCLIC1
Iba-1
TNF-α
Cleaved IL-1β
Pro-IL-1β
CD16
Arg1
IL-10
β-actin
Iba-1
15kDa
TNF-α
15kDa
Cleaved-
IL-1β
15kDa
35kDa
Pro-IL-1β
CD16
50kDa
Arg1
35kDa
IL-10
20kDa
β-actin
50kDa
40kDa

Figure 3N

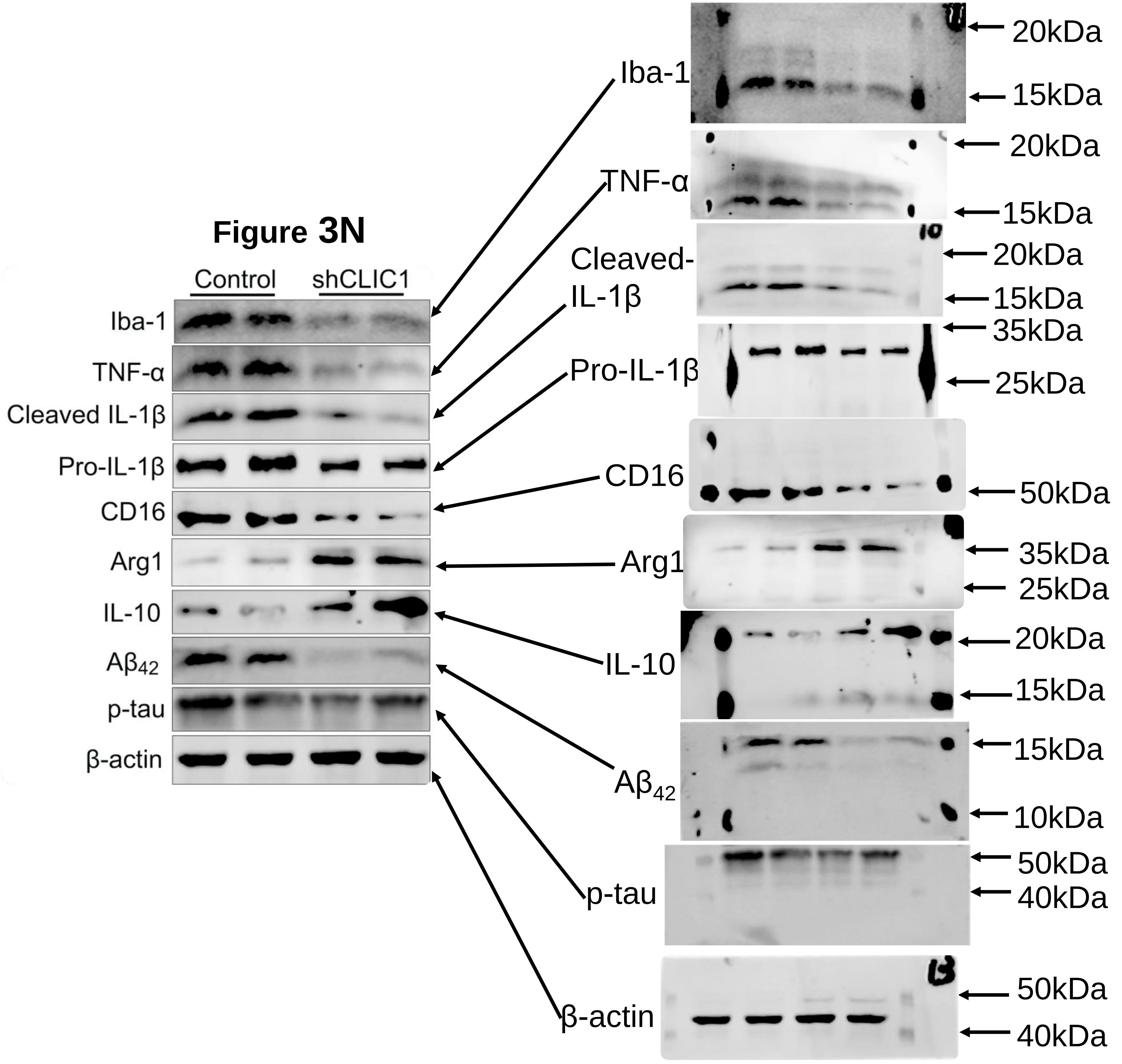
Control
shCLIC1
Iba-1
TNF-α
Cleaved IL-1β
Pro-IL-1β
CD16
Arg1
IL-10
Aβ42
p-tau
β-actin
Iba-1
20kDa
15kDa
TNF-α
20kDa
15kDa
Cleaved-IL-1β
20kDa
15kDa
Pro-IL-1β
35kDa
25kDa
CD16
50kDa
Arg1
35kDa
25kDa
IL-10
20kDa
15kDa
Aβ42
15kDa
10kDa
p-tau
50kDa
40kDa
β-actin
50kDa
40kDa

Figure 4G

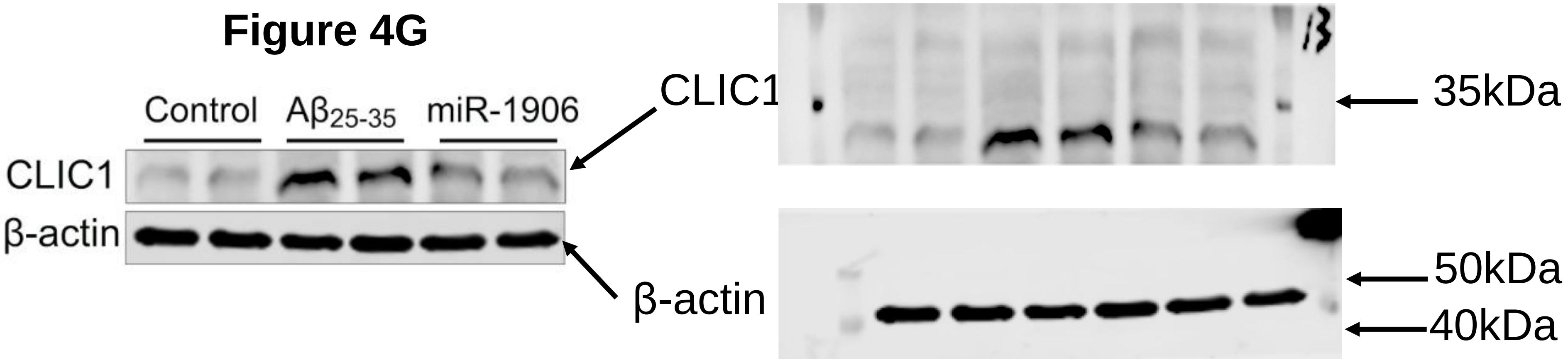

Figure 4H

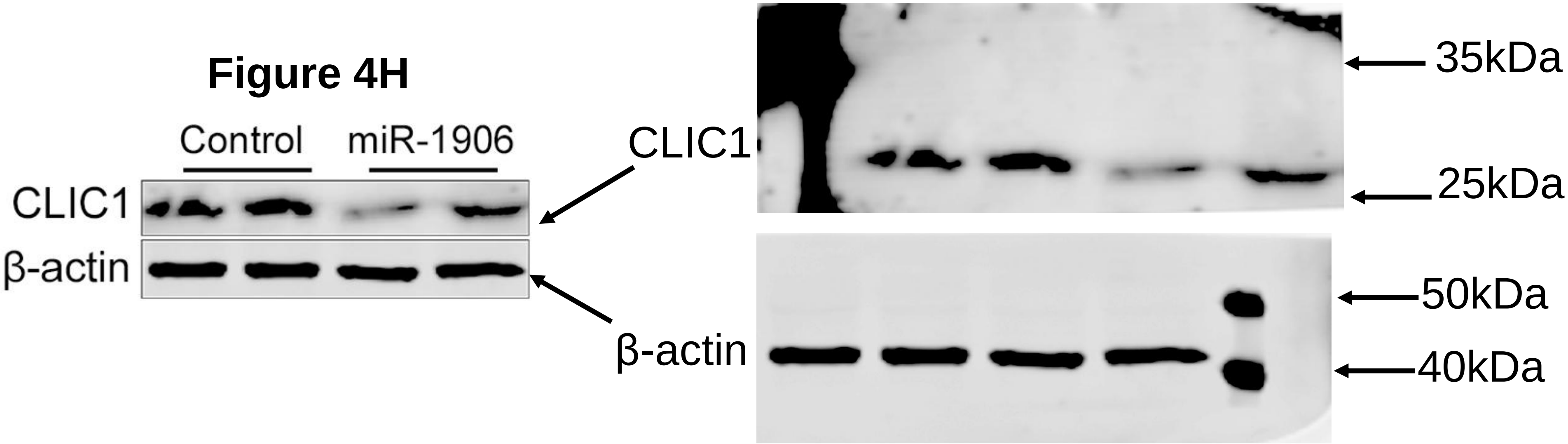

Figure 4I

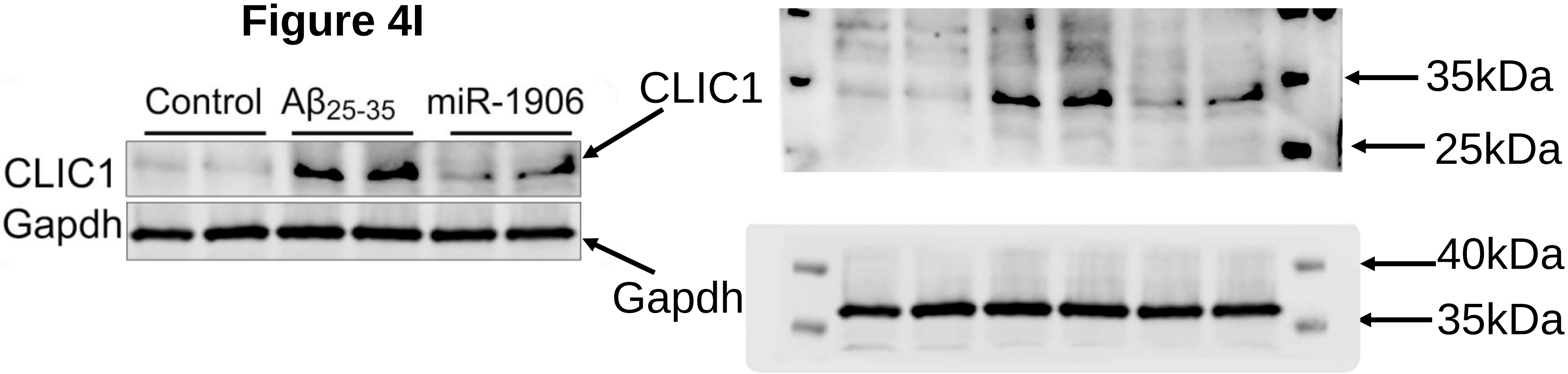

Figure 4K

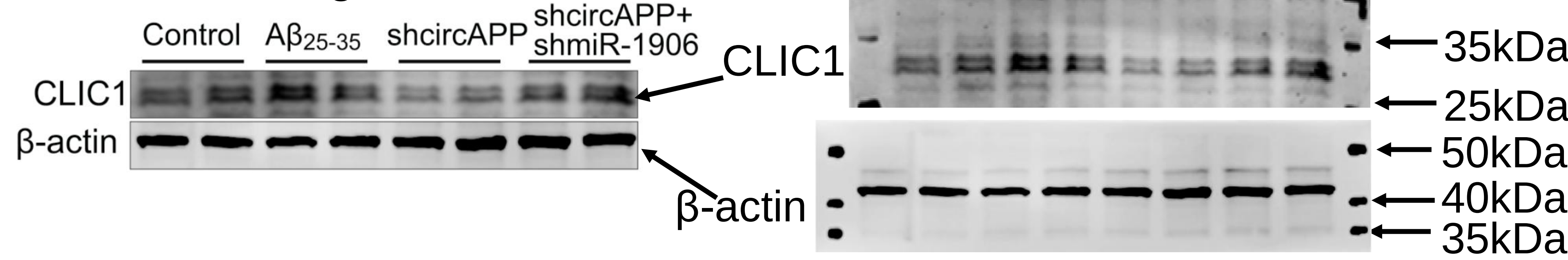

Figure 4L

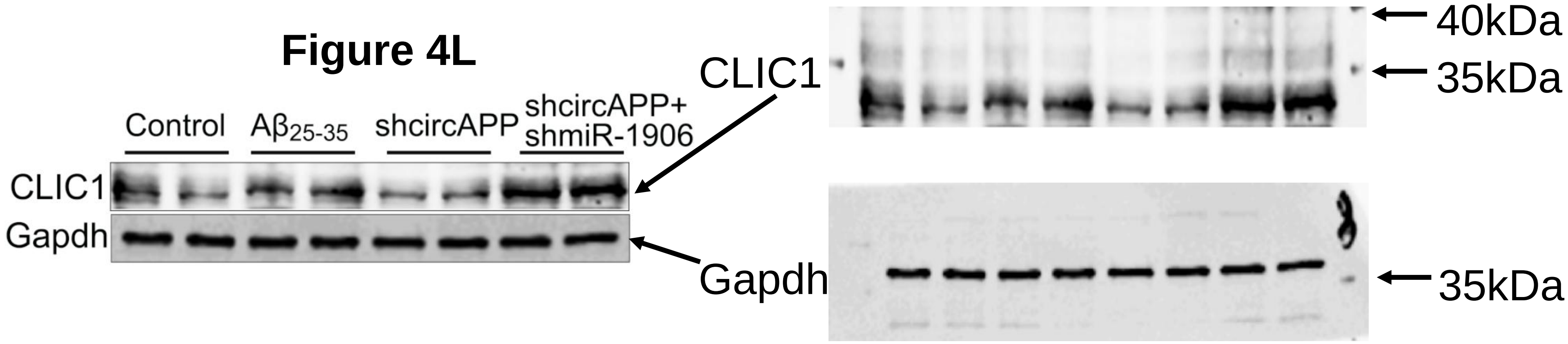

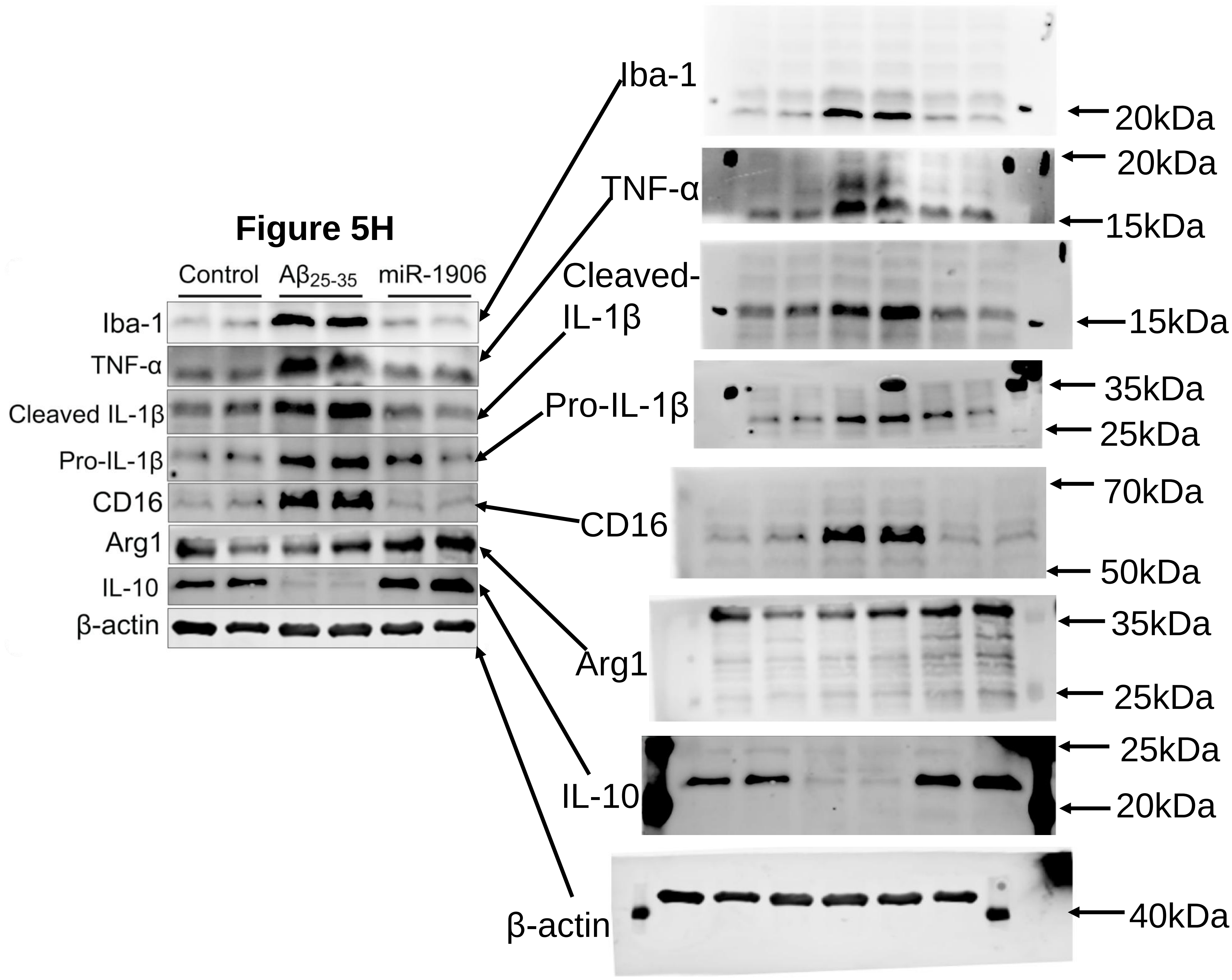

Figure 5H
Control
Aβ25-35
miR-1906
Iba-1
TNF-α
Cleaved IL-1β
Pro-IL-1β
CD16
Arg1
IL-10
β-actin
Iba-1
20kDa
TNF-α
20kDa
15kDa
Cleaved-IL-1β
15kDa
Pro-IL-1β
35kDa
25kDa
CD16
70kDa
50kDa
Arg1
35kDa
25kDa
IL-10
25kDa
20kDa
β-actin
40kDa

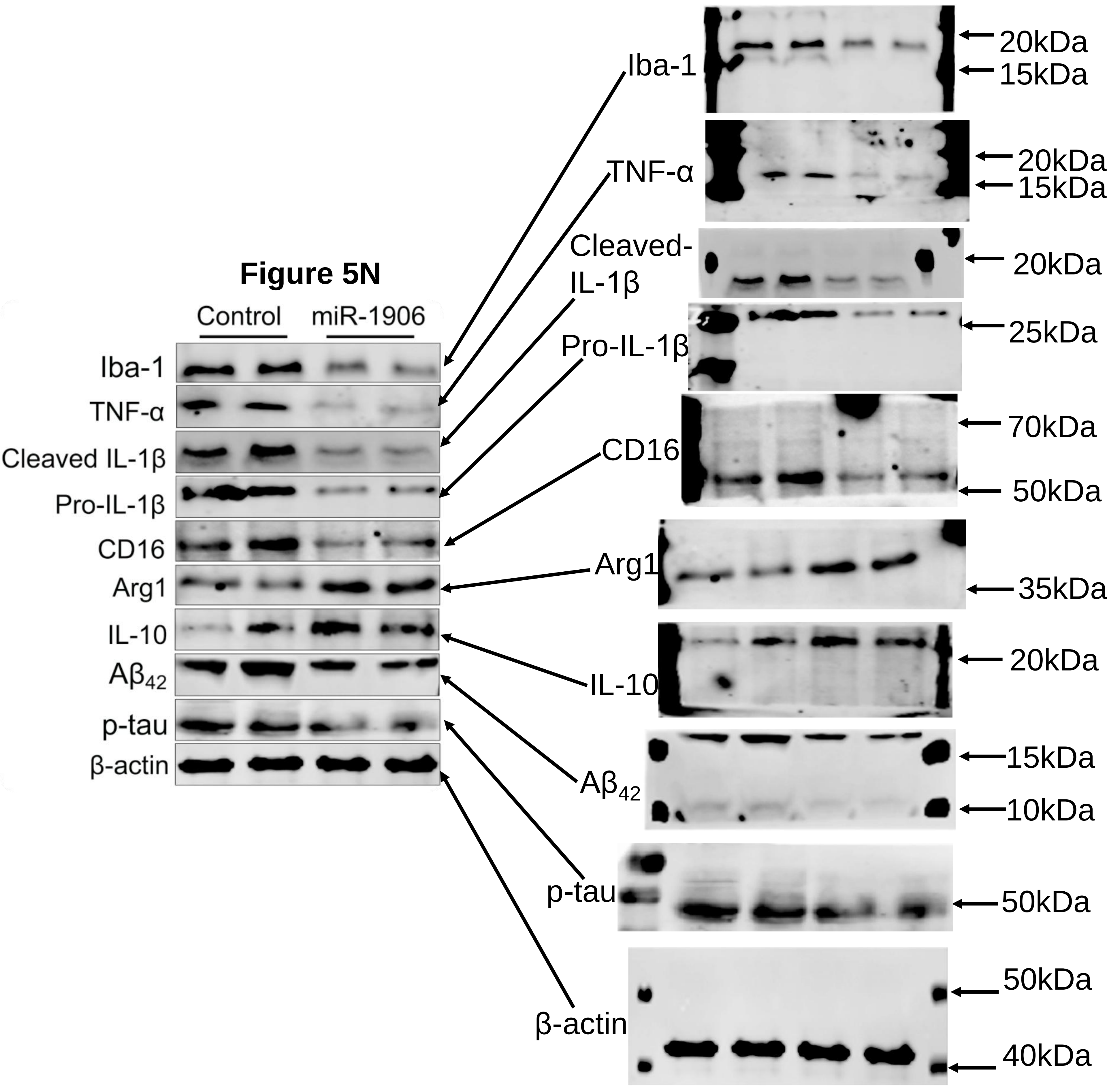

Figure 5N
Control
miR-1906
Iba-1
TNF-α
Cleaved IL-1β
Pro-IL-1β
CD16
Arg1
IL-10
Aβ42
p-tau
β-actin
Iba-1
20kDa
15kDa
TNF-α
20kDa
15kDa
Cleaved-IL-1β
20kDa
Pro-IL-1β
25kDa
CD16
70kDa
50kDa
Arg1
35kDa
IL-10
20kDa
Aβ42
15kDa
10kDa
p-tau
50kDa
β-actin
50kDa
40kDa

Figure 6A

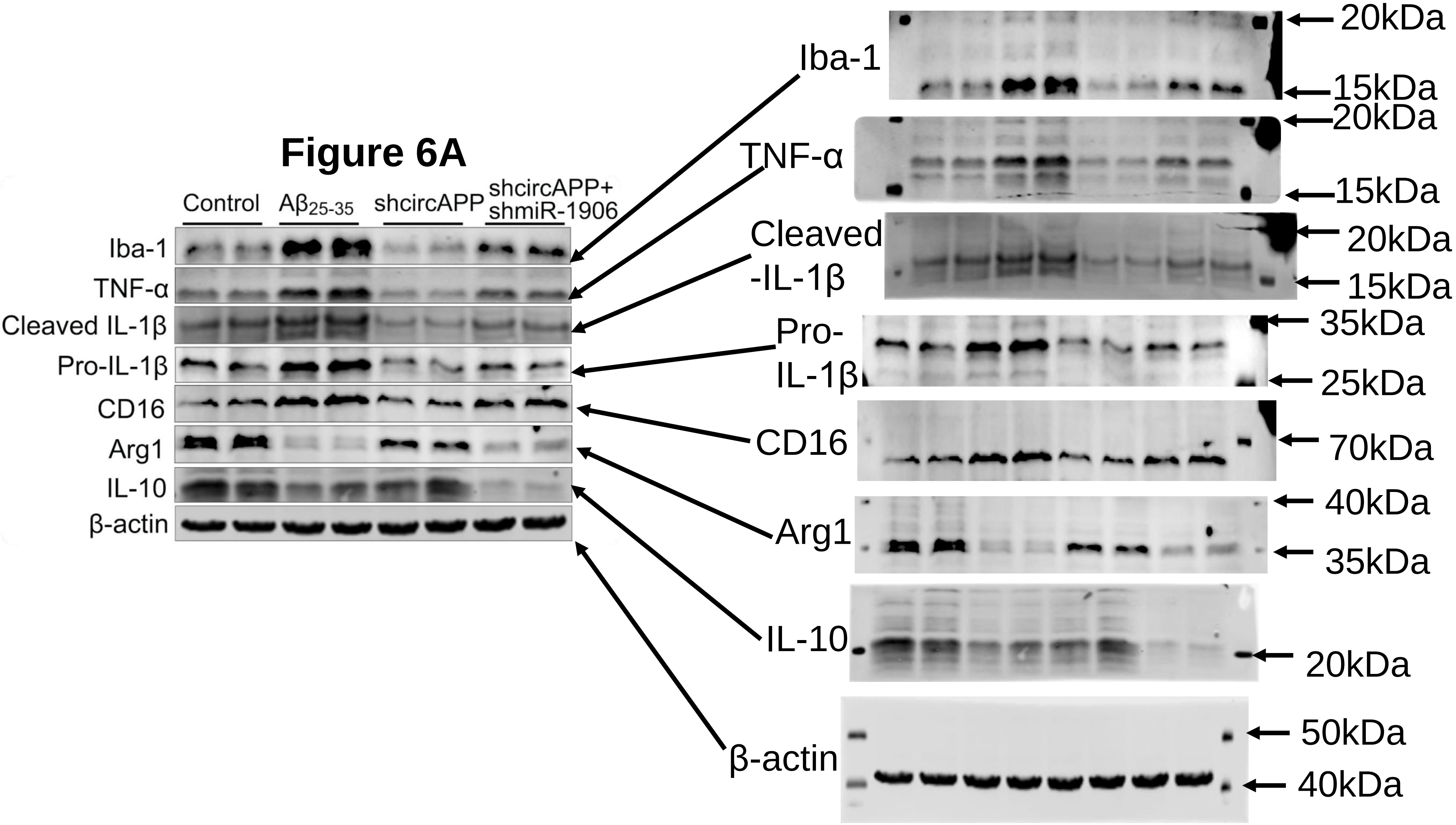

Control
Aβ25-35
shcircAPP
shcircAPP+ shmiR-1906
Iba-1
TNF-α
Cleaved IL-1β
Pro-IL-1β
CD16
Arg1
IL-10
β-actin
Iba-1
20kDa
15kDa
TNF-α
20kDa
15kDa
Cleaved -IL-1β
20kDa
15kDa
Pro-IL-1β
35kDa
25kDa
CD16
70kDa
Arg1
40kDa
35kDa
IL-10
20kDa
β-actin
50kDa
40kDa

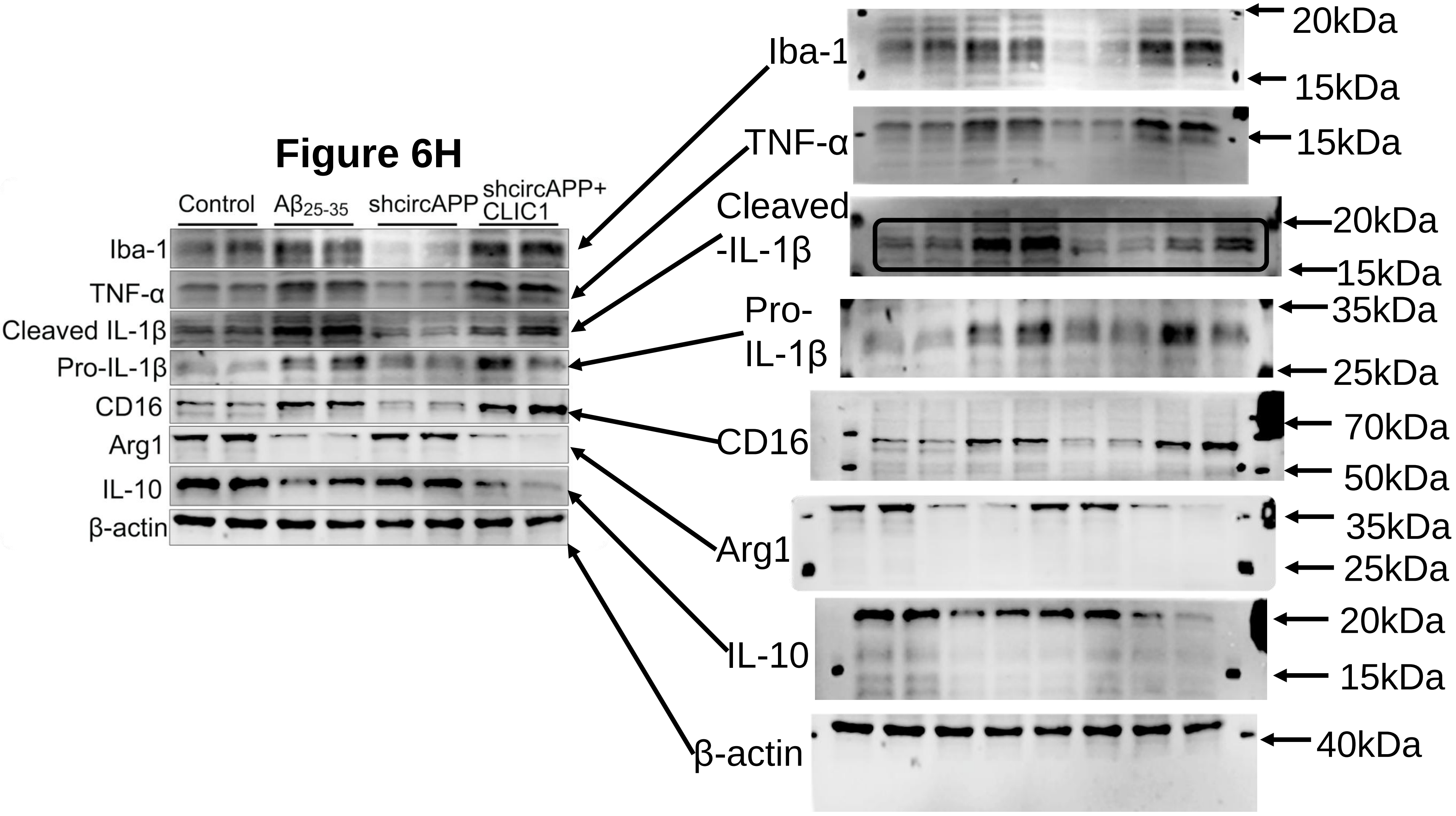
Figure 6H
Control
Aβ25-35
shcircAPP
shcircAPP+ CLIC1
Iba-1
TNF-α
Cleaved IL-1β
Pro-IL-1β
CD16
Arg1
IL-10
β-actin
Iba-1
20kDa
15kDa
TNF-α
15kDa
Cleaved -IL-1β
20kDa
15kDa
Pro-IL-1β
35kDa
25kDa
CD16
70kDa
50kDa
Arg1
35kDa
25kDa
IL-10
20kDa
15kDa
β-actin
40kDa

Figure 6O

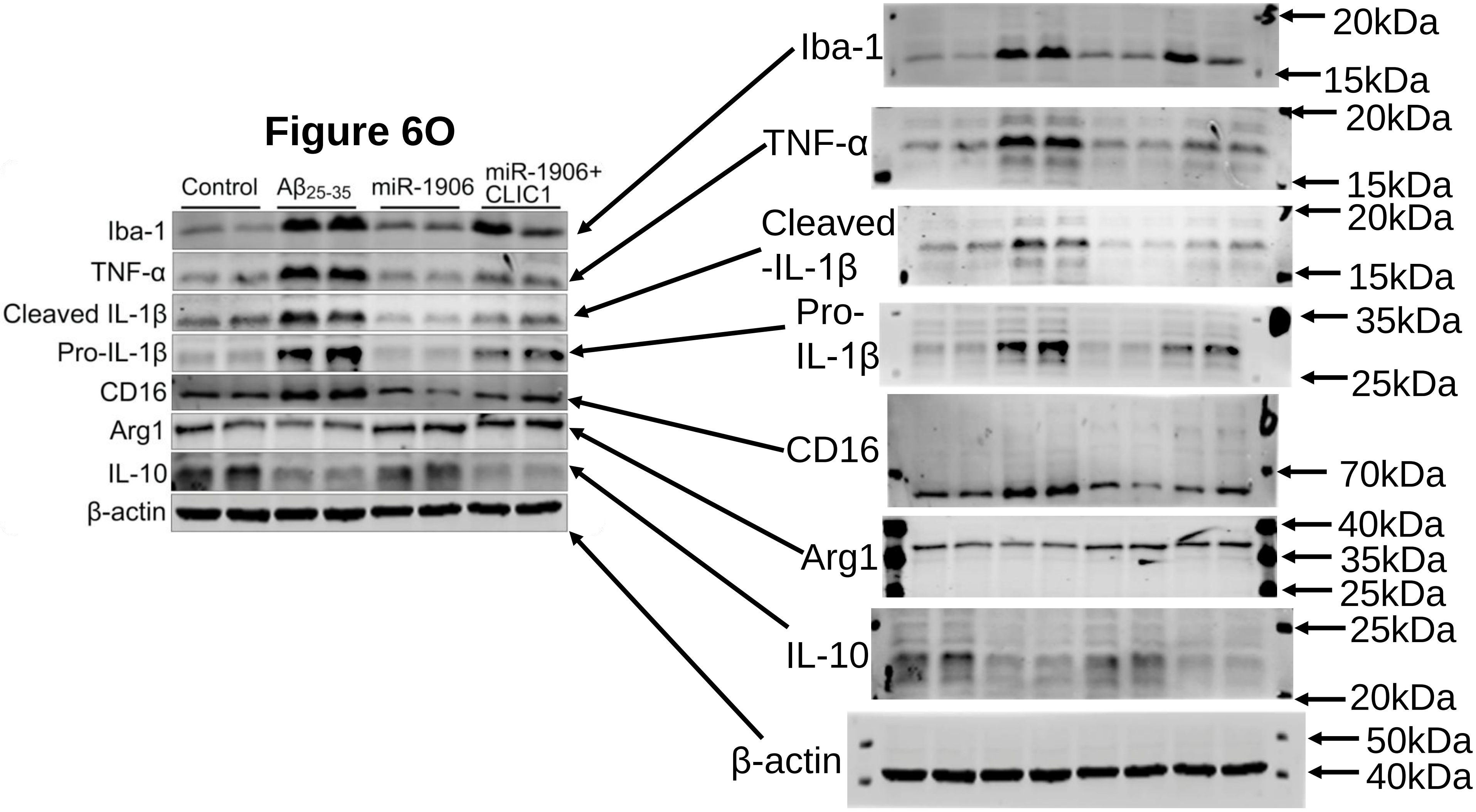

Control
Aβ25-35
miR-1906
miR-1906+ CLIC1
Iba-1
TNF-α
Cleaved IL-1β
Pro-IL-1β
CD16
Arg1
IL-10
β-actin
Iba-1
20kDa
15kDa
TNF-α
20kDa
15kDa
Cleaved -IL-1β
20kDa
15kDa
Pro-IL-1β
35kDa
25kDa
CD16
70kDa
Arg1
40kDa
35kDa
25kDa
IL-10
25kDa
20kDa
β-actin
50kDa
40kDa
